# Non-peptidic Cruzain Inhibitors with Trypanocidal Activity Discovered by Virtual Screening and In Vitro Assay

**DOI:** 10.1371/journal.pntd.0002370

**Published:** 2013-08-22

**Authors:** Helton J. Wiggers, Josmar R. Rocha, William B. Fernandes, Renata Sesti-Costa, Zumira A. Carneiro, Juliana Cheleski, Albérico B. F. da Silva, Luiz Juliano, Maria H. S. Cezari, João S. Silva, James H. McKerrow, Carlos A. Montanari

**Affiliations:** 1 Departamento de Química, Universidade Federal de São Carlos, São Carlos, São Paulo, Brazil; 2 Grupo de Química Medicinal do IQSC/USP, Instituto de Química de São Carlos, Universidade de São Paulo, São Carlos, São Paulo, Brazil; 3 University of California San Francisco, Center for Discovery and Innovation in Parasitic Diseases (CDIPD), Department of Pathology, San Francisco, California, United States of America; 4 Departamento de Bioquímica e Imunologia, Faculdade de Medicina de Ribeirão Preto, Universidade de São Paulo, Ribeirão Preto, São Paulo, Brazil; 5 Departamento de Biofísica, Escola Paulista de Medicina, Universidade Federal de São Paulo, São Paulo, Brazil; Instituto de Investigaciones Biotecnológicas, Argentina

## Abstract

A multi-step cascade strategy using integrated ligand- and target-based virtual screening methods was developed to select a small number of compounds from the ZINC database to be evaluated for trypanocidal activity. Winnowing the database to 23 selected compounds, 12 non-covalent binding cruzain inhibitors with affinity values (*K*_i_) in the low micromolar range (3–60 µM) acting through a competitive inhibition mechanism were identified. This mechanism has been confirmed by determining the binding mode of the cruzain inhibitor Nequimed176 through X-ray crystallographic studies. Cruzain, a validated therapeutic target for new chemotherapy for Chagas disease, also shares high similarity with the mammalian homolog cathepsin L. Because increased activity of cathepsin L is related to invasive properties and has been linked to metastatic cancer cells, cruzain inhibitors from the same library were assayed against it. Affinity values were in a similar range (4–80 µM), yielding poor selectivity towards cruzain but raising the possibility of investigating such inhibitors for their effect on cell proliferation. In order to select the most promising enzyme inhibitors retaining trypanocidal activity for structure-activity relationship (SAR) studies, the most potent cruzain inhibitors were assayed against *T. cruzi*-infected cells. Two compounds were found to have trypanocidal activity. Using compound Nequimed42 as precursor, an SAR was established in which the 2-acetamidothiophene-3-carboxamide group was identified as essential for enzyme and parasite inhibition activities. The IC_50_ value for compound Nequimed42 acting against the trypomastigote form of the *Tulahuen lacZ* strain was found to be 10.6±0.1 µM, tenfold lower than that obtained for benznidazole, which was taken as positive control. In addition, by employing the strategy of molecular simplification, a smaller compound derived from Nequimed42 with a ligand efficiency (LE) of 0.33 kcal mol^−1^ atom^−1^ (compound Nequimed176) is highlighted as a novel non-peptidic, non-covalent cruzain inhibitor as a trypanocidal agent candidate for optimization.

## Author Summary

Chagas disease (American trypanosomiasis) is a parasitic infection that kills millions of mostly poverty-stricken people in Latin America. In recent years it has also spread to nonendemic countries – the United States, Canada, Europe, Australia and Japan – as a result of immigration. The only available drugs for its treatment were introduced more than forty years ago, have low efficacy, and cause various severe side effects. This dire public health situation has prompted us to search for new small molecules to act as drug candidates to treat Chagas disease. The *T. cruzi* enzyme cruzain, a key biological catalyst used by the protozoan to digest host proteins, is a validated drug target for Chagas disease. By combining *in silico* molecular design, X-ray crystallography and biological screening, we found a new class of non-covalent small molecules that inhibit cruzain in low micromolar concentrations.

## Introduction

Chagas disease, widespread in Latin America, is caused by the kinetoplastid protozoan parasite *Trypanosoma cruzi*. Despite efforts to reduce the transmission of the parasite by controlling the hematophagous triatomine insect vector, the World Health Organization estimates that *10 million people* are *infected* worldwide, with another 25 million at risk. Most cases are in *Latin America*, where Chagas disease is endemic, but it is also found in Canada, the United States, Europe (mainly in Spain and Portugal), Japan and Australia [1]–[3].

*T. cruzi*'s complex life cycle involves two replicative forms: the epimastigote, in the gut of the insect vector, and the amastigote, an intracellular form in the infected mammal. The two infective non-replicative forms are the metacyclic trypomastigote in the insect vector and the bloodstream trypomastigote released from infected cells into the blood of the mammal [4].

Chagas disease has an acute phase and a chronic latent phase. The acute phase, which occurs shortly after infection, lasts for a few weeks or months, whereas the chronic phase develops over many years. The acute phase may not be noticed because it is symptom-free or exhibits only mild symptoms that are not unique to Chagas disease. These can include fever, fatigue, body aches, headache, and rash, loss of appetite, diarrhea and vomiting. Chronic phase symptoms appear between 10 and 20 years after infection and affect the heart, nervous and digestive systems.

The treatment of Chagas disease involves the front-line drugs nifurtimox and benznidazole. These two old drugs are effective at curing the infection mostly in the acute phase, with successful cure up to 80%, but are almost ineffective in chronically infected patients [5]. Moreover, due to its collateral effects nifurtimox is no longer available in most Latin American countries. In addition to the severe side effects of the available chemotherapy, drug-resistance has been observed in some trypanosome strains. Thus, the discovery of new, safer and more effective drugs to treat Chagas disease is of utmost importance.

Cruzain (a recombinant form of cruzipain, EC 3.4.22.51) has excellent pre-clinical validation evidence as a druggable target. Cruzain belongs to the family of cysteine proteases (papain-like enzymes known as clan CA) and is closely related to cathepsins L and S, which are also associated with other pathologies in humans [6]. It is the major cysteine protease in *T. cruzi* and is essential for the development and survival of the parasite within the host cells. Numerous protease inhibitors with different scaffolds and catalytic mechanisms show activity against the parasite in culture and animal models of the disease [7]. Clan CA cysteine proteases are effectively inhibited by several classes of peptide inhibitors including transition state-based, reversible and irreversible inhibitors [8]. Examples of reversible transition state-based inhibitors are peptide aldehydes, α-diketones, α-ketoesters, α-ketoamides and α-ketoacids [9]. Clan CA proteases are also irreversibly inhibited by peptidyldiazomethyl ketones, fluoromethyl ketones, peptide epoxides (E-64, E-64-c, E-64-d) and vinyl sulfones [10]. Recently, non-covalent inhibitors have been discovered through high-throughput screening (HTS) platforms and, despite their lower potency relative to previously reported covalent compounds, they represent important breakthroughs in the development of non-peptidic compounds with drug-like features [11], [12].

**Figure 1:**
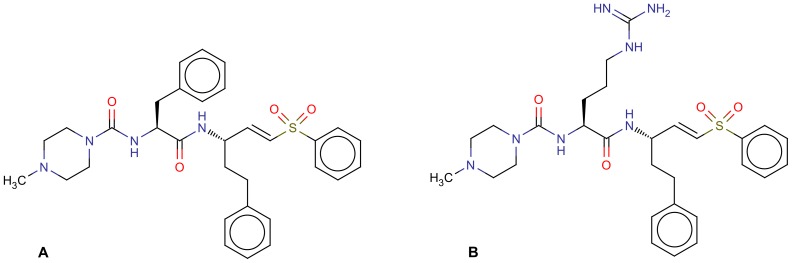
2D structural representation of (A) K11777 and (B) WRR-483 inhibitors.

A promising molecular class acting with antiparasitic activity can be found in vinyl sulfones. In pre-clinical trials, the inhibitor K11777 (Figure 1A) has been shown to be non-mutagenic, well tolerated, to have an acceptable pharmacokinetic profile and demonstrated efficacy in models of acute and chronic Chagas disease both in mice and dogs [13]. Additional studies of vinyl sulfone compounds have led to the identification of an arginine variant of K11777, named WRR-483 (Figure 1B) with remarkable biological properties [14].

The aim of this study was to identify new molecular classes of cruzain inhibitors by focusing on non-peptidic non-covalent ligands. To this end, we have carried out virtual screening of the ZINC Database [15], a free database of commercially-available compounds for virtual screening, utilizing ligand- and target-based virtual screening methods [16], [17], followed by enzymatic assays, X-ray crystallography and SAR studies of the most promising hits. Of nine cruzain inhibitors, five show trypanocidal activity against the trypomastigote infective form of the *Tulahuen lacZ* strain. We also expect that a newly identified fragment of the 2-acetamidothiophene-3-carboxamide class can advance the search for new non-covalent cruzain inhibitors.

## Methods

### Computational methods

**Figure 2:**
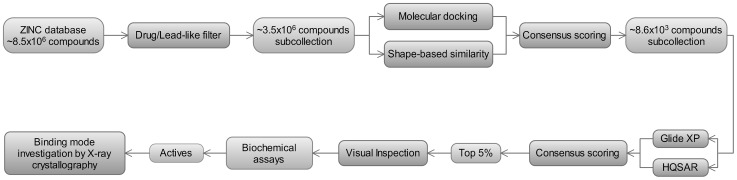
A scheme of the multi-step virtual screening protocol used for the identification of cruzain inhibitors.

A variety of methods are available to virtually screen small organic compound databases. A multi-step cascade strategy using integrated ligand- and target-based virtual screening methods was applied as illustrated in Figure 2.

#### Ligand-based methods

FILTER (v2.0.2): The FILTER program (OpenEye Scientific Software) [18] was used to filter ca. 8.5 million chemical structures in the ZINC version 8.0 database. This molecular filtering tool uses a combination of physical property calculations and functional group properties to assess libraries and ultimately remove non drug/lead-like compounds. The default drug-like parameter settings were modified in order to accommodate known cruzain inhibitors. Parameters modified were: ?? molecular weight (minimum value = 300 Da, maximum value = 700 Da), number of heavy atoms (15–35 atoms), number of ring systems (0–5), number of functional groups (0–18), number of connected unbranched non-ring atoms (0–6), number of carbons (7–45), number of heteroatoms (2–20), halide fraction (0–6), number of rotatable bonds (2–20), number of rigid bonds (0–35), number of Lipinski violations 2. Predicted known aggregators and compounds of moderate to low calculated solubility were excluded.

OMEGA (v2.0.2): Compounds that passed through FILTER were assembled into a conformer library using the OMEGA program [19]–[21]. The algorithm implemented in OMEGA dissects molecules into fragments and reassembles them to generate many possible conformations, then submits each conformer to a simplified energy evaluation. Next, all conformers below a defined energy threshold are compared and those falling within a certain root mean square deviation of atomic coordinates (RMSD) are clustered into a single representative group. Default parameters were used with the following exceptions: (1) ewindow (a parameter used to discard high-energy conformations), set to 25.0 kcal mol^−1^; (2) maxconfs (maximum number of conformations to be generated), set to 500 (default = 400). This library of conformers was employed as input to the ROCS and FRED programs.

ROCS (v2.4.1): ROCS (Rapid Overlay of Chemical Structures, OpenEye) uses a shape-based superposition method in which molecules are aligned by maximizing the overlap volume between a reference structure (the query molecule) and every conformer of the molecules contained in the database. The ligands K11777 (D1R, PDB ID 2OZ2) and T10 (PDB ID 1ME4) in co-crystallized conformations retrieved from X-ray structures deposited in the Protein Data Bank, were used as query molecules. The degree of structural similarity was calculated using ComboScore, the sum of the Shape Tanimoto and the Scaled Color values. This choice was made based on previous findings where this metric showed good performance in retrieving cruzain inhibitors from a dataset composed of active and inactive compounds [22]–[25].

HQSAR: A Quantitative Structure Activity Relationships Based on Molecular Hologram model had been previously developed and validated [24]. Since p*K_i_* value calculations are fast and the dataset is independent of conformation and alignments, this HQSAR model was used in a prospective way in order to prioritize compounds for *in vitro* assays.

#### Target-based methods

The 3D structure of cruzain (PDB ID 1ME4) used for docking was prepared with the Biopolymer structure preparation module from Sybyl v8.0 (Tripos International). Ligand and water molecules were subtracted and all missing hydrogen atoms were added to the protein structure. The residues belonging to the catalytic triad Cys25 and His159 were set as uncharged and the azo-hydrogen of His159 was kept on the delta nitrogen, since structural data show the epsilon nitrogen as a possible hydrogen bond acceptor from the hydroxyl group of the α-hydroxy-ketone ligand. The prepared structure was used as the initial state for molecular docking in all docking programs.

FRED (v2.2.5) [26]: Default docking parameters were used with the following exceptions: inner and outer contour maps were disabled, so that the van der Waals radii of active site atoms and box limits were taken as thresholds for docking. The box was added to encompass all residues within 5 Å from the ligand coordinates to represent the site for docking. Consensus scoring, with PLP, chemgauss3 and oechemscore scoring functions were enabled during selection of the pose and, along with all remaining available functions (shapegauss, chemgauss2, chemscore, screenscore and zapbind) were also used to score the selected pose. Predicted binding energy for each of these scoring functions was used in the analysis. The Gly66 Nα (H-bond donor) and Leu67 side chain (hydrophobic positional) were set as interaction constraints in order to achieve a RMSD <2 Å in the re-docking experiment.

Glide (v4.5) [27], [28]: As input for the program Glide (Schrödinger), the receptor grid generation was generated within a grid box of 30×30×30 Å^3^, centered on the complexed ligand coordinates. Gly66 Nα (H-bond donor) and Leu67 side chain (hydrophobic positional) were set as interaction constraints; all remaining parameters for docking were kept in the default mode. The extra precision scoring function (Glide XP) was used to score the docked compounds. These inputs were sufficient to predict a crystallographic pose of the complexed ligand with RMSD <2 Å in the re-docking experiment

### Biochemical assays

#### Enzyme assays

Recombinant cruzain enzyme was expressed and purified as previously described [29]. The activity of the cruzain enzyme was measured and quantified through active-site titration with the irreversible inhibitor E-64, as described previously [30]. Cathepsin L enzyme and all reagents used for buffer preparation were purchased from Sigma-Aldrich (St. Louis, MO, USA). The compounds screened against the enzymes were purchased from Enamine (Kiev, Ukraine), Chemdiv (San Diego, CA, USA), Asinex (Moscow, Russia) and IBScreen (Moscow, Russia) and had more than 95%purity, according to these suppliers. All experiments were carried out with freshly made solutions.

The activity of cruzain was measured in 100 mM phosphate buffer, 100 mM NaCl, 10 mM EDTA, DMSO (5.0% v/v), Triton X-100 (0.01% v/v) and 5.0 mM DTT at pH 6.3. The activity of cathepsin L was measured in 100 mM sodium acetate buffer, 100 mM NaCl, 10 mM EDTA, DMSO (5.0% v/v), Triton X-100 (0.01% v/v) and 5.0 mM DTT at pH 5.5. Both assays were carried out with 2 nM of the respective enzymes and 2 µM of the substrate Z-FR-MCA.

#### IC_50_ determination

IC_50_ measurements were carried out by incubating the enzyme with the buffer containing DTT for activation of the cruzain for 5 minutes, followed by the addition of inhibitors in concentrations varying from 150 µM to 0.15 µM with additional 5 minutes incubation. The reaction started by the addition of the substrate Z-FR-MCA was monitored by recording the emission at wavelength 460 nm while exciting at 360 nm. Fluorescence was measured using a *Synergy HT* fluorimeter microplate reader (Biotek). The percentage of inhibition was calculated according to following equation:
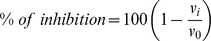
where *v_i_* and *v_0_* are the initial velocities determined in presence and absence of inhibitors, respectively. The activity of enzyme in the presence of inhibitors was measured in 11 different concentrations in order to obtain the dose-response curves, which were analyzed using the Origin 7.0 program to determine the IC_50_ values. All experiments were repeated at least in duplicate.

#### Affinity constant determination

The Michaelis-Menten curves were obtained for affinity constant (K*_i_*) determinations. The buffer solutions used for cruzain and cathepsin-L enzymes were the same as for the activity assays, with the exception that substrate concentration was changed from 0.1 to 10 µM.

The Michaelis-Menten curves were determined in the absence and presence of 30, 50 and 70 µM respectively of each inhibitor. The enzymes were incubated for 5 minutes with buffer containing DTT followed by 5 minutes incubation with inhibitors. The reaction was started and monitored as described above. The mechanisms of inhibition and *K*_i_ values were determined using the kinetic module of *SigmaPlot* 10.0 by adjusting the velocities into the competitive, non-competitive and uncompetitive modes [31]. All experiments were done at least in duplicate.

#### Crystal structure of cruzain co-complexed with compound Neq176

Recombinant cruzain was expressed and purified in a modified version as described recently by Lee *et al.* 2012 [32]. The 0.5 mg mL^−1^ solution of procruzain (in 100 mM sodium acetate, 10 mM EDTA and 300 mM NaCl, at pH 5.2) was activated at 37°C for 3.5 hours with 5 mM DTT. After activation, cruzain was immediately inhibited with the covalent reversible inhibitor methyl methanethiosulfonate (MMTS) to a final concentration of 1 mM to prevent self-degradation of the protein. The protein was dialyzed in 50 mM Tris, 300 mM NaCl pH 7.5, and concentrated to 3 mg mL^−1^ for purification using preparative scale Superdex 200 (10/300), size exclusion column (GE Healthcare Life Sciences) and the same buffer (50 mM Tris, 300 mM NaCl pH 7.5), with flow 0.5 ml min^−1^. The fractions with the pure MMTS-inhibited cruzain (highest peak at 34 min) were collected, concentrated to 8 mg mL^−1^, and buffer-exchanged in 2 mM Bis-Tris pH 5.8. MMTS was reversibly removed with 5 mM of DTT. The inhibitor Neq176, the one with higher ligand efficiency, was added until the final concentration of 4.2 mM (1.5% of DMSO) was attained with the final ratio of 1∶8.5 (protein/inhibitor). The solution was stirred for 1.5 hours at 4°C until protein inhibition was complete. The protein was then concentrated to 11 mg mL^−1^. Hanging drops encompassing 192 crystallographic conditions (Joint Center Structure Genomics screens III–IV, Qiagen) were configured using a Mosquito Nanoliter Dropsetter (TTP Labtech). Each condition was screened in 1∶1 and 2∶1 ratio between protein solution and mother liquor. After one week of incubation at 20°C, crystals were obtained in 0.1 M Bicine pH 9.0, 1.6 M ammonium sulfate (100 nL protein solution):(100 nL mother liquor) drops. Crystals were then reproduced under the same conditions in 2 µL hanging drops. Before data collection, crystals were immersed in a cryoprotectant solution composed of 25% ethylene glycol in mother liquor, and flash cooled in liquid nitrogen.

Diffraction was measured at beamline 8.3.1 of the Advanced Light Source, (ALS, Lawrence Berkeley Lab, CA), using ELVES [33] to determine the data collection strategy. The best crystal of the series diffracted at 2.62 Å resolution. Reflections were indexed and integrated using Mosflm [34] and scaled using SCALA [35]. The initial phasing model without waters, ligands, and heteroatoms used for molecular replacement in Phaser [36] was prepared from the model (PDB entry 3KKU). Phenix Refine [37] and Coot [38] were used for all steps of structure refinement and model building. The model was positioned initially by rigid body refinement and subjected to one round of simulated annealing to reduce model bias with torsional non-crystallographic symmetry, followed by multiple cycles of individual coordinate refinement and B_factor_ refinement. B-factors were refined isotropically and the protein was subjected to TLS refinement.

The geometry of the structure was assessed using Molprobity [39]. There were no outliers in the Ramachandran statistics, with 97.3% of all residues in favored regions. The data collection and refinement statistics can be found in Table 1.

**Table 1:** Data collection and refinement statistics.

*Data collection*	
Wavelength (Å)	1.116
Space group	P 4_3_ 2_1_ 2
No. of molecules in ASU	5
Cell dimensions (Å)	
a, b, c (Å)	138.72 138.72 163.58
α, β, γ (°)	90 90 90
*R_merge_ (%)*	2.6 (33.5)^a^
Completeness (%)	99.5 (98.4)^a^
*I/σ(I)*	15.9 (2.1)^a^
Redundancy	2.6 (2.0)^a^
**Refinement**	
Resolution (Å)	39.23 - 2.62 (2.71 - 2.62)^a^
No. of reflections (test set)	48282 (1954)^a^
*R_work_/R_free_ (%)*	18.99/23.89
No. atoms	
*Protein* b	7960
Ligand^b^	54
*Water*	109
*B-factors* (Å^2^)	
*Protein*	33.50
*Ligand*	84.00
*Water*	26.30
*R.m.s. deviations*	
*Bond lengths* (Å)	0.008
*Bond angle (°)*	1.08

a Values in parenthesis represents the highest resolution shells.

b Calculated for both molecules in the asymmetric units.

#### In vitro evaluation of trypanocidal activity

*In vitro* trypanocidal activity of the compounds was evaluated against amastigote forms of Tulahuen strain that was genetically modified to express β-galactosidase gene from *E. coli* (lacZ). A monkey kidney cell strain (LLC-MK2 - ATCC) was resuspended in RPMI medium without phenol red (Gibco-BRL Life Technologies, Grand Island, NY) containing 10% fetal bovine serum (Life Technologies Inc., Bethesda, MD) and antibiotics (Sigma Chemical Co., St. Louis) at 2×10^3^ cells/well and were cultured in 96-well plates for 24 h. The cells were infected with 1×10^4^ trypomastigote forms of *T. cruzi* Tulahuen strain, and after 24 h compounds were added with different concentrations (250, 125, 62.5, 31.25, 15.6, 7.8, 3.9 and 1.95 µM). After 4 days of culture, 50 µL of PBS containing 0.5% of Triton X-100 and 100 µM Chlorophenol Red-β-D-galactoside (CPRG - Sigma) were added. Plates were incubated at 37°C for 4 h and absorbance was read at 570 nm. Benznidazole (*N*-benzyl-2-nitro-1-imidazolacetamide), in the same concentrations as above was used as a reference trypanocidal drug (positive control) [40], [41].

#### Cytotoxicity assays

Mammalian cell cytotoxicity was evaluated using the protocol previously reported [42]. Spleen cells from C57BL/6 mice were isolated by mechanical dissociation, followed by incubation for 5 min with red blood cell lysis buffer (one part of 0.17 M Tris–HCl [pH 7.5] and nine parts of 0.16 M ammonium chloride). The cells were washed and suspended in RPMI 1640 medium (Gibco-BRL Life Technologies, Grand Island, NY) supplemented with 10% fetal bovine serum (Life Technologies Inc., Bethesda, MD) and antibiotics (Sigma). The cell suspension was cultured in flat-bottom 96-well plates at 5×105 cells per well with different concentrations of the tested compounds and incubated at 37°C in a humidified atmosphere of 5% CO2 for 24 h. Tween 20 at 0.5% was used as cell death positive control. To analyze cytotoxicity, cells were harvested, incubated with 10 µg mL^−1^ propidium iodide (Sigma) and after 15 min data were acquired using a FACSCantoII (Becton-Dickinson Immunocytometry System Inc., San Jose, CA, USA). Data analysis was performed using FlowJo software (Ashland, OR, USA). The range of concentrations used for assaying cytotoxicity was 250, 125, 62.5, 31.25, 15.6, 7.8, 3.9, and 1.95 (µM).

### Ethics statement

The Ethics Committee on Animal Experimentation of the Faculty of Medicine of Ribeirão Preto – University of Sao Paulo approved the cytotoxicity assays (approval no. 076/2010). This Committee adheres to Conselho Nacional de Controle de Experimentação Animal – CONCEA, created by Brazilian Law number 11794 of 8 October 2008. Assays were run according to the guidelines of the Ministry of Science, Technology and Innovation of Brazil.

## Results and Discussion

The compound collection enrichment process began by the filtering ca. 8.5 million structures from the ZINC Database, resulting in a sub-library containing ca. 3.5 million structures. Since the sub-library still encompassed a large number of structures, two fast virtual screening methods based on ligand and receptor were employed in order to enrich it. The agreement between ligand- and target-based virtual screening methods was then used as the criterion for the selection of an enriched, focused sub-library. Based on the predicted Tanimoto similarity metric and docking score of known inhibitors, a set of thresholds was established for the selection of untested compounds. The values adopted as thresholds and the numbers of compounds resulting from this analysis are summarized in Table 2.

**Table 2:** Thresholds for FRED and ROCS adopted for initial enrichment of compound library and number of compounds resulting from each VS experiment.

Method (scoring function/metric)	Threshold	Number of compounds retrieved
FRED (SHAPEGAUSS)	−470	16151
ROCS query 1 (ComboScore)	0.71	9090
ROCS query 2 (ComboScore)	0.68	6159

The analysis of cruzain-inhibitor complexes available in the PDB database reveals the importance of residues Gly66 and Leu67, located between subsites S2 and S3, to the molecular recognition of ligands. The amidic nitrogen of Gly66 along with the amidic oxygen of Asp158 frequently interact via hydrogen bonding with amides from known cruzain inhibitors. Furthermore, Leu67 is able to accommodate hydrophobic groups that occupy the S2 portion of the active site, thus contributing to adequate shape-matching of small molecules in this cleft. Hence, we have used these important interactions to set Gly66 and Leu67 as constraints for pose selection in all docking experiments. Based on these constraints and established thresholds, the molecular docking using the FRED program performed well at discriminating between active and inactive compounds when using the SHAPEGAUSS score function. Although the binding energy predicted by the molecular docking programs is not rigorously calculated, it was used as preliminary filter for lessening the number of molecules in the compound library.

As can be seen in Table 2, the thresholds established from available information of known ligands and employed as criteria for selecting compounds in the virtual screening (VS) experiments resulted in a significant enrichment of the compound collection. Since compounds retrieved according to the metric threshold adopted for each program may differ, we used this lack of agreement for the removal of more compounds from the enriched collection. Only those compounds retrieved by both programs that had a docking score or similarity index above the defined threshold were selected to join the smaller final sub-collection. Thus, the integration of target- and ligand-based methods resulted in the selection of a series of compounds with high potential to inhibit the enzyme cruzain and with diverse chemical features to allow the identification of new scaffolds to target this enzyme.

The reduction of the number of molecules in the virtual library allowed us to apply the consensus score strategy including p*K_i_* values calculated by the HQSAR (Hologram Quantitative Structure Activity Relationship) model and Glide Extra Precision scoring function (Glide XP), which requires a higher computational cost compared to the ROCS and FRED programs. Combined ranking was calculated using the scaled rank-by-number approach as described in [25]; the top 5% compounds were submitted to visual inspection.

**Figure 3:**
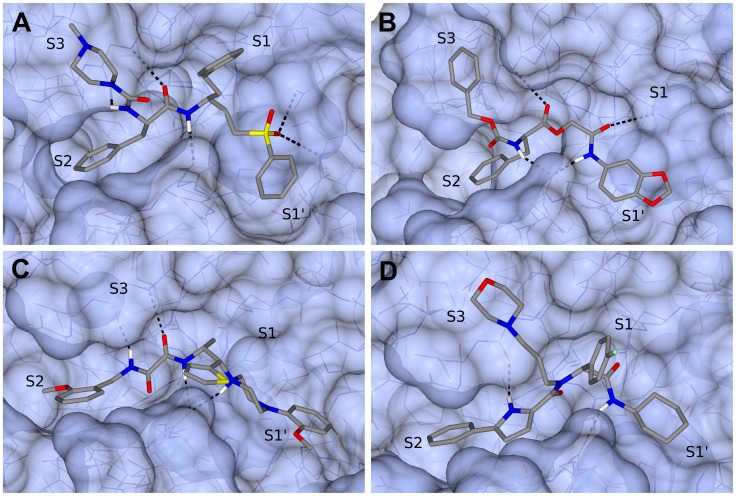
(A) Structure of the co-crystallized cruzain inhibitor K11777 and (B–D) examples of complex structures predicted by our molecular docking (Glide XP). Figure prepared using CCP4mg software [43].

The criteria used for selection of compounds based on visual inspection were: (i) the occupation of the site, mainly in subsites S1, S2 and S3, (ii) hydrogen bonding, emphasizing the Gly66 H-bonding interaction set up as constraint in molecular docking and (iii) chemical structure diversity, where we prioritized compounds with similar shape to known inhibitors. Figure 3A shows the binding mode of K11777 inhibitor to the enzyme cruzain in the crystal structure conformation, which was also used as reference in the 3D similarity search; Figure 3B to Figure 3D are the docking poses predicted by Glide XP for three compounds selected for *in vitro* assays. As can be seen, the occupation of the active site by these compounds is similar to that of the co-crystalized inhibitor, appropriately filling the focused pockets and fulfilling the requirements imposed through H-bonding constraint. [43]

**Figure 4:**
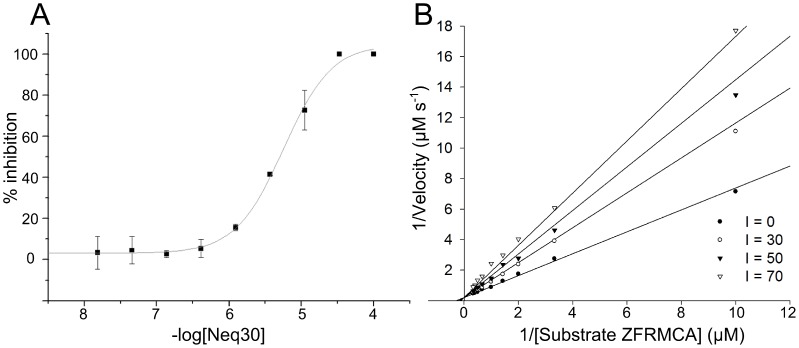
(A) Dose-response and (B) Linewaver-Burk curves for Neq30 from Table S1. Non-linear fit method was employed in the analysis.

After visual inspection, 23 compounds were selected for *in vitro* assays against the enzyme cruzain. First, the activity of compounds was determined through measurement of IC_50_ values in the presence of Triton X-100 (0.01% v/v) in order to avoid artifactual aggregate-based inhibition. Afterwards, those compounds that showed activities had their mechanism of enzyme inhibition and affinity constants determined. Dose-response curves were used for measuring the IC_50_ values and Michaelis-Menten curves in the presence and absence of inhibitors in three different concentrations, which allowed the determination of the mechanism of inhibition and affinity constants. Since the identified active compounds showed similar curves, representative curves are shown in Figure 4A and 4B for Neq30 of Table S1 (see Supporting Information). A similar approach for the discovery of cruzain inhibitors is described elsewhere by Ferreira *et al.* (see reference [44]). Using the docking strategy the authors were able to find one inhibitor out of 17 screened compounds that displayed a *K*_i_ of 32 µM, via a competitive mechanism of cruzain inhibition. Our consensus ligand-based virtual screening (LBVS) and target-based virtual screening (TBVS) approaches also using the Lineweaver-Burk plots in a similar fashion confirmed the competitive mode of action of 12 out of 23 compounds whose average IC_50_ value is 40.3 µM (with 3 compounds in the range of 3.5 µM) – see Table S1.

The dose-response plot in Figure 4A shows the inflexion of the semi-log curve fit with a pIC_50_ of 5.12 µM, which corresponds to the potency exhibited by Neq30 listed in Table S1. As shown in Figure 4B the increase in the concentration of inhibitor causes a decrease in the affinity of substrate but no change in the maximum velocity, thus indicating that the inhibitor competes for the same site as substrate Z-FR-MCA.

Cruzain shares high similarity with the mammalian homolog cathepsin L. Thus, it is of interest to evaluate selectivity [45] of selected compounds on both these enzymes, since promising trypanocidal scaffolds that act on cruzain could also be relevant to target cathepsin L, which has been studied as target for the treatment of cancer. Therefore, these compounds were tested against both enzymes. The 2D molecular structure representations of assayed compounds, IC_50_ and *K_i_* values are shown in Table S1 of the Supporting Information.

The multi-step virtual screening protocol designed to identify cruzain inhibitors was validated by the discovery of 12 hits with affinity to the enzymes ranging from 3.7 to 89 µM among only 23 compounds assayed in the *in vitro* enzymatic assay. Recent studies report the search for cruzain inhibitors in the ZINC database using virtual screening methods. Ferreira *et al*. selected 17 compounds using molecular docking and found one active compound with an IC_50_ of 77 µM, which was further optimized by SAR to yield an IC_50_ of 200 nM [44]. Using a ligand-based virtual screening approach, Malvezzi *et al*. found one compound with low micromolar potency among 19 selected by a pharmacophore model [46]. By comparison, the number of hits we found using virtual screening methods was higher, which can be attributed to the integrated TBVS and LBVS consensual strategy. Consensus strategies and multi-step cascade protocols have been shown to achieve higher hit-rates when compared to LBVS or TBVS used separately [25], [47]–[49]. In order to confirm the activity of the scaffold found we carried out an initial SAR using the approach called SAR by catalog. This is a particularly powerful (and generally accessible) approach to the initial development of fragments [50]–[52], which assists in progressing through scaffolds with different R-groups.

**Figure 5:**
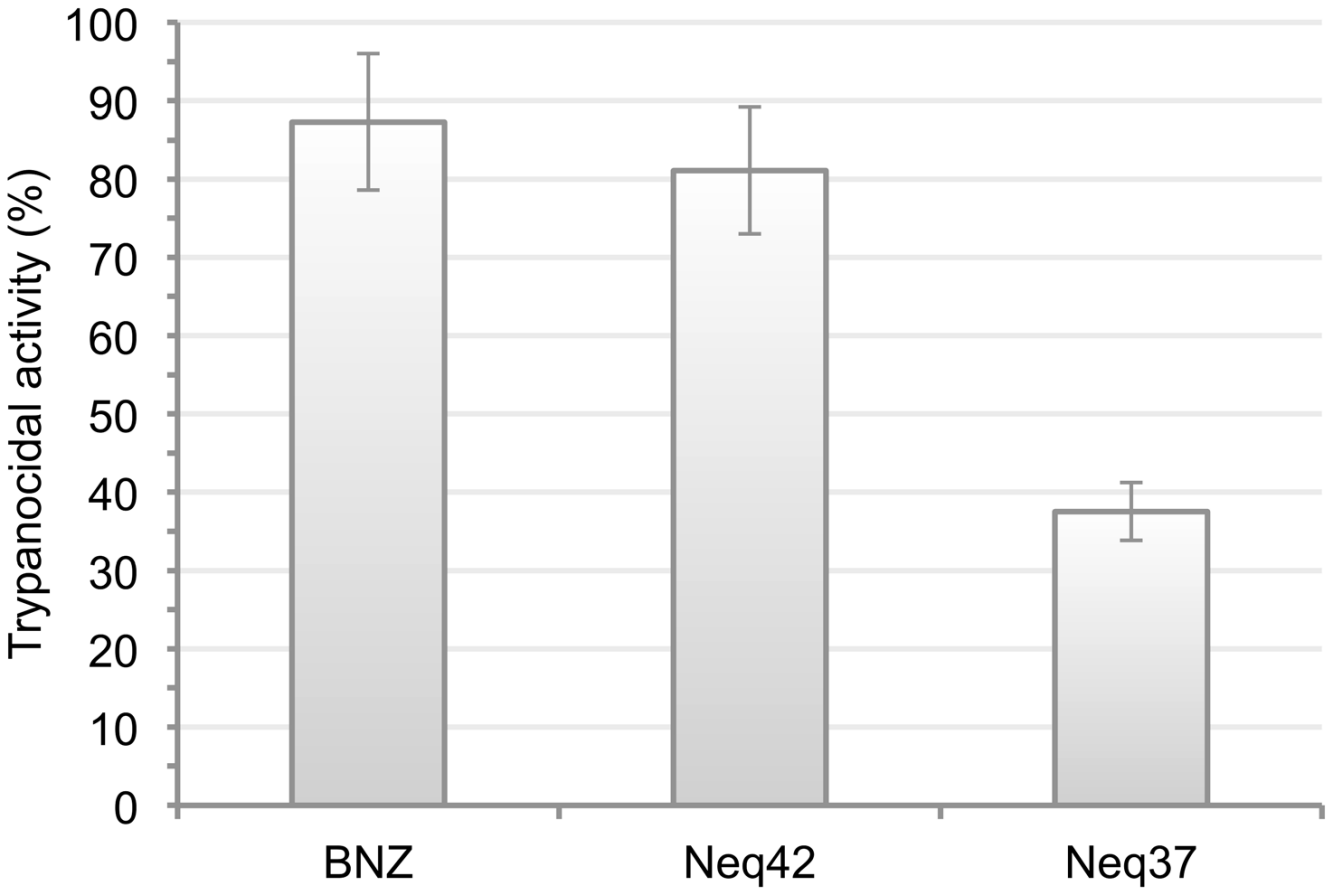
Preliminary trypanocidal activity of compounds Neq42 and Neq37 evaluated against the Tulahuen lacZ strain.

The establishment of peptide SAR is often the first step in defining a critical, minimum sequence SAR for modulation of a particular target. The convenient and rapid synthesis of peptide analogs facilitates the identification of peptides having attractive biological properties. However, peptidomimetics possess well-recognized burdens as potential drugs, including susceptibility to enzymatic or chemical hydrolysis of peptide bonds and the metabolism of amino acid side chains, which influences the investigator in favor of discovering non-peptidic scaffolds with drug-like properties for cruzain inhibitors. Therefore, among the most potent compounds, there are new non-peptidic scaffolds including the compounds Neq24, Neq25, Neq30 and Neq42, for instance. Thus, before hit optimization, we evaluated trypanocidal activity of the six most potent compounds with the aim of selecting the most promising for molecular optimization. This assay was carried out at a single dose of 250 µM using the same procedure described above. Figure 5 shows the results for the two active compounds Neq42 and Neq37.

Compound Neq42, which represents a new scaffold for non-peptidic cruzain inhibitors, was able to kill the *T. cruzi* parasite and displayed similar activity to benznidazole, which was used as positive control in the assays. For this reason, we have chosen this compound as a reference to investigate its SAR.

Due to the moderate molecular complexity of the compound Neq42, a structure simplification strategy was adopted for SAR investigation taking as reference not only its potency but also its ligand efficiency (LE), a way of normalizing the potency and MW of a ligand to provide a useful comparison between compounds with a range of MWs and activities. The higher the LE of a hit, the better its chance of being optimized to a potent drug-like compound [53], [54].

**Figure 6:**
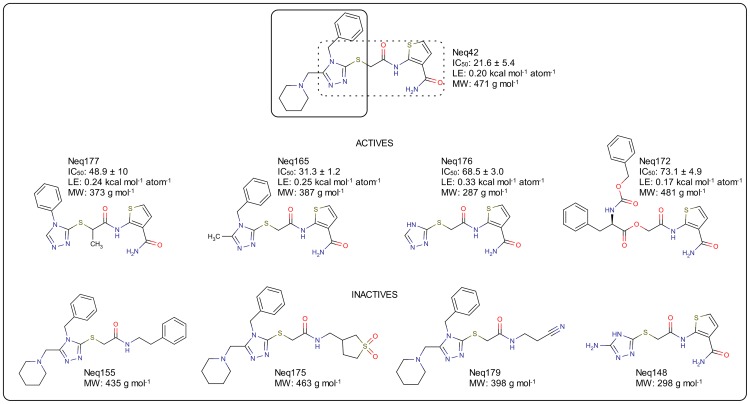
Molecular structure and cruzain inhibition of compound Neq42 analogs selected for SAR investigation. Molecules drawing and figure generated with MarvinSketch software (www.chemaxon.com).

The structure of Neq42 was resolved into two main scaffolds: the first containing the 2-acetamidothiophene-3-carboxamide moiety, and the other the triazole ring moiety substituted in positions 1 and 2 with the benzyl and piperidine, respectively, as can be seen in Figure 6. Based on the predicted mode of binding obtained by molecular docking, a series of structures was selected from commercial databases to investigate the SAR of this compound (Figure 6). When the 2-acetamidothiophene-3-carboxamidegroup was maintained, the piperidine and benzyl groups were removed to assess their contribution to the potency. On the other hand, when the triazole moiety was kept, three substances were selected by replacing the 2-acetamidothiophene-3-carboxamide group to a phenyl group (hydrophobic), a nitrile (which might covalently bind to the catalytic cysteine) and a cyclic sulfone (hydrophilic). The 2D structures and biological activity of the selected compounds are summarized in Figure 6.

**Figure 7:**
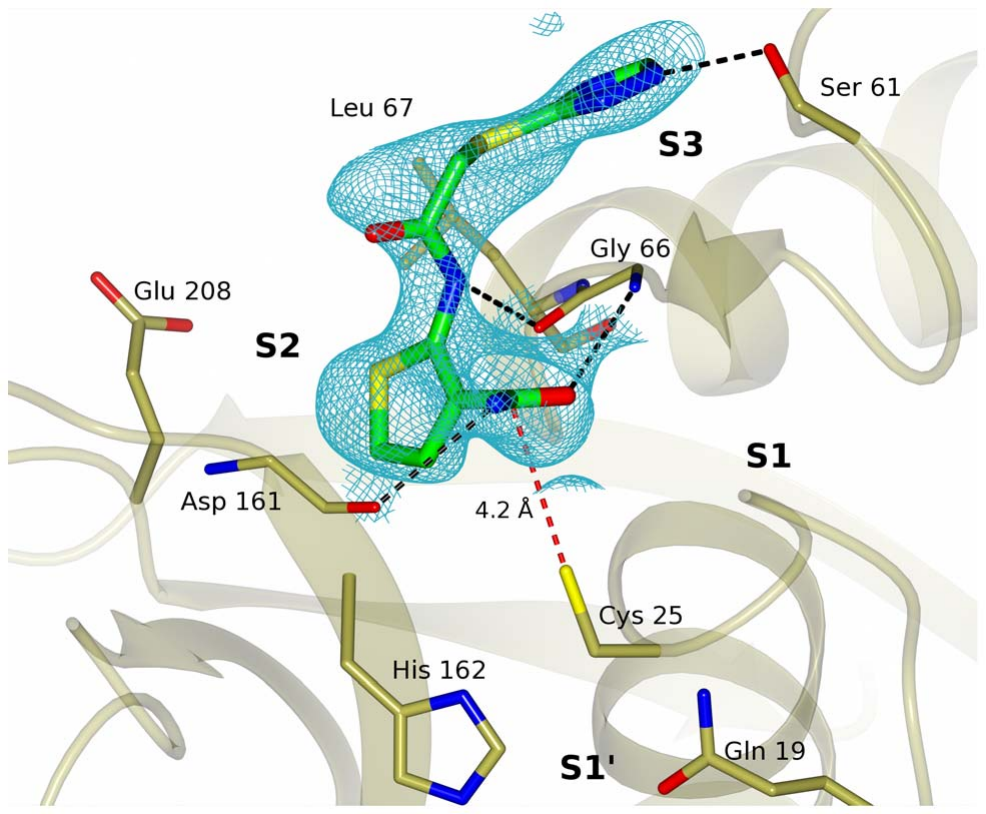
Crystal structure of Neq176 co-crystallized with cruzain showing the mode of binding (MOB) of the inhibitor at the catalytic site of chain B with the unbiased mFo-DFc electron density map shown in cyan. Figure prepared using CCP4mg software [43].

 The 2-acetamidothiophene-3-carboxamide is probably the one responsible for the activity presented by compound Neq42. As can be observed by the comparison between the active and inactive series shown in Figure 6, molecules lacking this moiety completely lose their activity against the cruzain enzyme. Nevertheless, potencies of compounds Neq165, Neq176, Neq177 are still in a similar order of magnitude as compound Neq42, notwithstanding a significant MW lowering that results in an increased LE for the analogs (0.33 kcal mol^−1^ atom^−1^ for compound Neq176). This observation signals that the piperidine and benzyl groups give rise only to a minor contribution to the potency, since removal of both groups (compound Neq176) resulted only in a slight decrease in potency, but with a significant LE improvement to 0.33 kcal mol^−1^ atom^−1^. The compound Neq172, which is a combination derived from compounds Neq38 and Neq42, resulted in smaller LE and potency, once again evincing the importance of 2-acetamidothiophene-3-carboxamide for the activity. Not only tailored LEs were substantially increased but also a new non-peptidic scaffold, which is an excellent starting point for optimization, was identified in agreement with the proposed binding mode shown in Figure 7. [43]

In order to evaluate the mode of binding (MOB) for Neq176, the crystallographic structure for cruzain-Neq176 complex was determined to 2.62 Å resolution. The protein crystallized at P4_3_2_1_2 space group with five copies of cruzain in the asymmetric unit, with the compound Neq176 bound in three of the copies (chains A, B and C) in a similar mode of binding. The Neq176 binds in the S2 and S3 pockets, with the 2-acetamidothiophene-3-carboxamide group making two hydrogen bond interactions with the Nα of Gly66 (3.15 Å) and with the alpha oxygen of Asp161 (3.08 Å). The N(2) atom of the inhibitor also makes an important H-bond interaction with the alpha oxygen of Gly66 (3.09 Å). The inhibitor is stabilized at the S3 site by the interaction of the N(4) atom from the 1,2,4-triazole group with the γ oxygen of Ser61 (2.67 Å). Crystal structure showing the binding mode (MOB) of Neq176 with cruzain wild type can be found in Figure 7. Similar hydrogen bonding pattern of interactions was previously recognized for a covalent inhibitor (PDB entry 3IUT) [55]. Nonetheless, our X-ray structural data analysis confirm that the fragment Neq176, albeit within the active site, is not covalently bound to native cruzain since the carbonyl center group in the thiophene moiety is 4.20 Å distant from Sγ of the Cys25. The coordinates and structure factors of the protein were deposited in Protein Data Bank (PDB) with the ID code 4KLB.

An important feature in these compounds is related to the mechanism of inhibition they disclose, which are competitive and reversible. The validation of these cruzain enzyme inhibitors, along with their innovative molecular class pinpoint these compounds as promising for the development of new trypanocidal agents. In order to further strengthen the molecular basis of these findings, active compounds shown in Figure 6 were assayed against cultures of *T. cruzi* parasite following the protocol described above. Results are summarized in Table 3.

**Table 3:** Trypanocidal activity and cytotoxicity of cruzain inhibitors evaluated against Tulahuen lacZ strain.

Compounds	IC_50_ (µM)	Cytotoxic IC_50_ (µM)
Bz	64.3±12.3	>500
Neq42	10.6±0.1	49.9±2.7
Neq177	137±14.4	>250
Neq165	153.4±0.6	N.d.
Neq176	108.3±52.2	>250
Neq172	166.1±12.9	23.1±2.7

Bz: benznidazole. N.d., not determined. See text for explanation.

As can be seen from Table 3, compound Neq42 analogs presented trypanocidal activity at micromolar range validating this molecular class not only as enzyme inhibitors, but also as trypanocidal agents (pIC_50_ ca. 3.8 on average for compounds Neq165, Neq172, Neq176, and Neq177, and 4.9 for compound Neq42 versus 4.2 for benznidazole). Furthermore, as observed for the activity against cruzain enzyme, the decoration in the triazole ring does not significantly contribute to the trypanocidal activity among the actives. This suggests that major contribution for activity arises from the 2-acetamidothiophene-3-carboxamide group, which put forward the idea that triazole replacement might be a crucial element for designing new compounds with improved potency.

Compounds Neq177 and Neq176 are active cruzain and *T. cruzi* inhibitors that are cytotoxic only at concentrations above 250 µM, whilst compounds Neq42 and Neq172 are cytotoxic at IC_50_ of 50 and 23 µM, respectively (Table 3). These values were construed by tailoring down the chemical complexity of compound Neq42, which yielded the percentage of cell death caused by compounds Neq176 and Neq177 as evaluated against the cultured mouse spleen cells to dramatically drop and to show a significant decrease in cytotoxicity values. Thus, although compounds Neq176 and Neq177 are the least cytotoxic trypanocidal agents toward the studied cells, their potencies toward *T. cruzi* decreased along with decreased potencies against cruzain. Compound Neq42, on the other hand, has the higher potency in this series against cruzain, paralleling its higher trypanocidal potency, but with increased cytotoxicity. Nevertheless, it is noteworthy that the cytotoxicity ratio (IC_50_(cyto)/IC_50_)(*T. cruzi*) for compound Neq42 and benznidazole is within the same magnitude range values (5 and 8, respectively), and thus these structure-activity and-toxicity relationships enlarge lead optimization opportunities.

In addition, it is known that compounds bearing the 2-acetamidothiophene-3-carboxamide moiety are also found to be inhibitors of IKKβ kinase phosphorylation of IκB, thus being inhibitors of NF-κB activation [56]. Cruzain knockout with inhibitors are also lethal for *T. cruzi* via the signaling factor NF-κB P65, which is colocalized with cruzain on the cell surface of the intracellular wild *T. cruzi* [57]. Hence, we may envisage that our compounds will be of interest in the search for new drug candidates acting on inflammatory cardiomyopathy that is a hallmark on Chagas disease, but acting as non-covalent inhibitors.

### Conclusions

Chagas disease is a neglected trypanosomiasis with enormous social and economic impact in most countries of Latin America. It is of utmost importance to develop new and more effective drugs with fewer side effects than the currently available chemotherapy. Hitherto, significant efforts have been made focusing on cruzain enzyme as a promising target and compound K11777, a cruzain inhibitor set to enter clinical studies as a new antichagasic drug. Here, we successfully used integrated *in silico* and *in vitro* approaches, with X-ray crystallography as an orthogonal tool, to discover new non-peptidic hits with trypanocidal activity against cruzain. Thus, we identified new trypanocidal agents that bear the 2-acetamidothiophene-3-carboxamide as the group responsible for enzyme inhibition and trypanocidal activity. The 2-acetamidothiophene-3-carboxamide binds non-covalently to cruzain, does not violate the rule of five and actually is a fragment with proper ligand efficiency (0.33 kcal mol^−1^ atom^−1^), with a low molecular mass (283.3 g mol^−1^) and CLogP of 0.7, properties that illuminate the way ahead for maneuvering toward a lead-like molecule.

In summary, we present a new hit, 2-acetamidothiophene-3-carboxamide, that non-covalently inhibits cruzain, has trypanocidal activity and manageable structure-activity and structure-toxicity relationships. We anticipate that this compound will advance the lead optimization process for Chagas disease chemotherapy.

### Further reading

Detailed information on cruzain EC 3.4.22.51 (also known as cruzipain) can be found at http://www.brenda-enzymes.org/php/result_flat.php4?ecno=3.4.22.51&Suchword=&organism%5B%5D=Trypanosomacruzi&show_tm=0 and also at https://www.ebi.ac.uk/chembldb/target/inspect/CHEMBL3563. For Cathepsin L, this site is also of interest: https://www.ebi.ac.uk/chembldb/target/inspect/CHEMBL3837.

## Supporting Information

Table S1 **2D structure representation,** ***K*** **_i_ and IC_50_ of the compounds assayed against cruzain and cathepsin L enzymes.**
(DOC)
