# Supplementary material for: Non-peptidic Cruzain Inhibitors with Trypanocidal Activity Discovered by Virtual Screening and In Vitro Assay
Source: PLoS Negl Trop Dis. 2013 Aug 22;7(8):e2370. doi: 10.1371/journal.pntd.0002370 (PMC3750009; doi:10.1371/journal.pntd.0002370)
Supplement: Table S1 — 2D structure representation, K i and IC50 of the compounds assayed against cruzain and cathepsin L enzymes. (DOC) [file pntd.0002370.s001.doc]

Table S1. 2D structure representation, *K*_i_ and IC_50_ of the compounds assayed against cruzain and cathepsin L enzymes.

| **Compound**  **ID** | **Structure** | **Cruzain**  ***K_i_* (µM)** | **Cathepsin L**  ***K_i_* (µM)** | **Cruzain IC_50_ (µM)** | **Cathepsin L IC_50_ (µM)** |
| --- | --- | --- | --- | --- | --- |
| Neq30 | 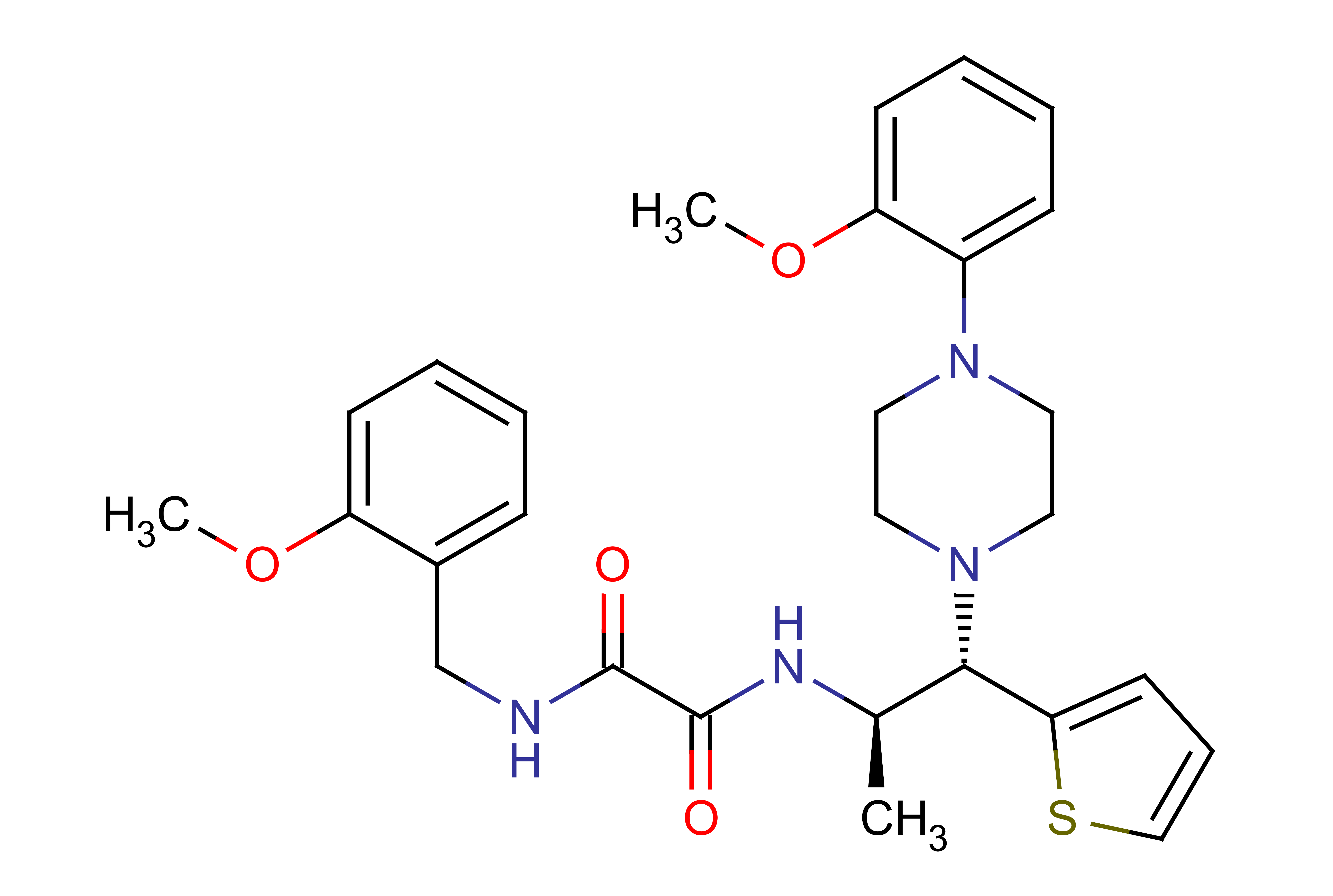 | 3.7 ± 0.4 | 16.7 ± 3.4 | 7.5 ± 2.3 | 9.9 ± 1.3 |
| Neq38 | 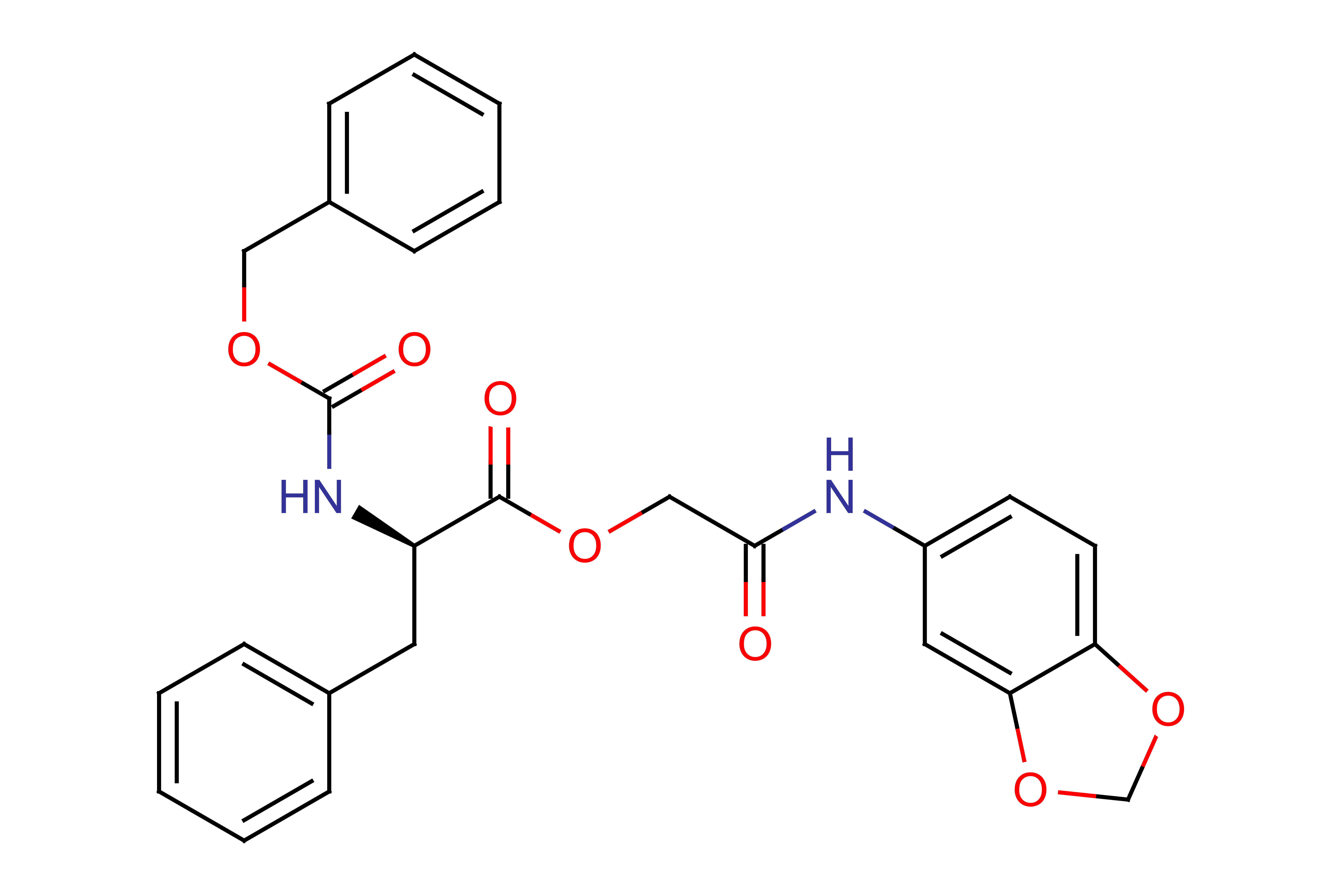 | 5.8 ± 1.6 | 31.1 ± 1.1 | 5.6 ± 1.1 | 33.1 ± 1.8 |
| Neq25 | 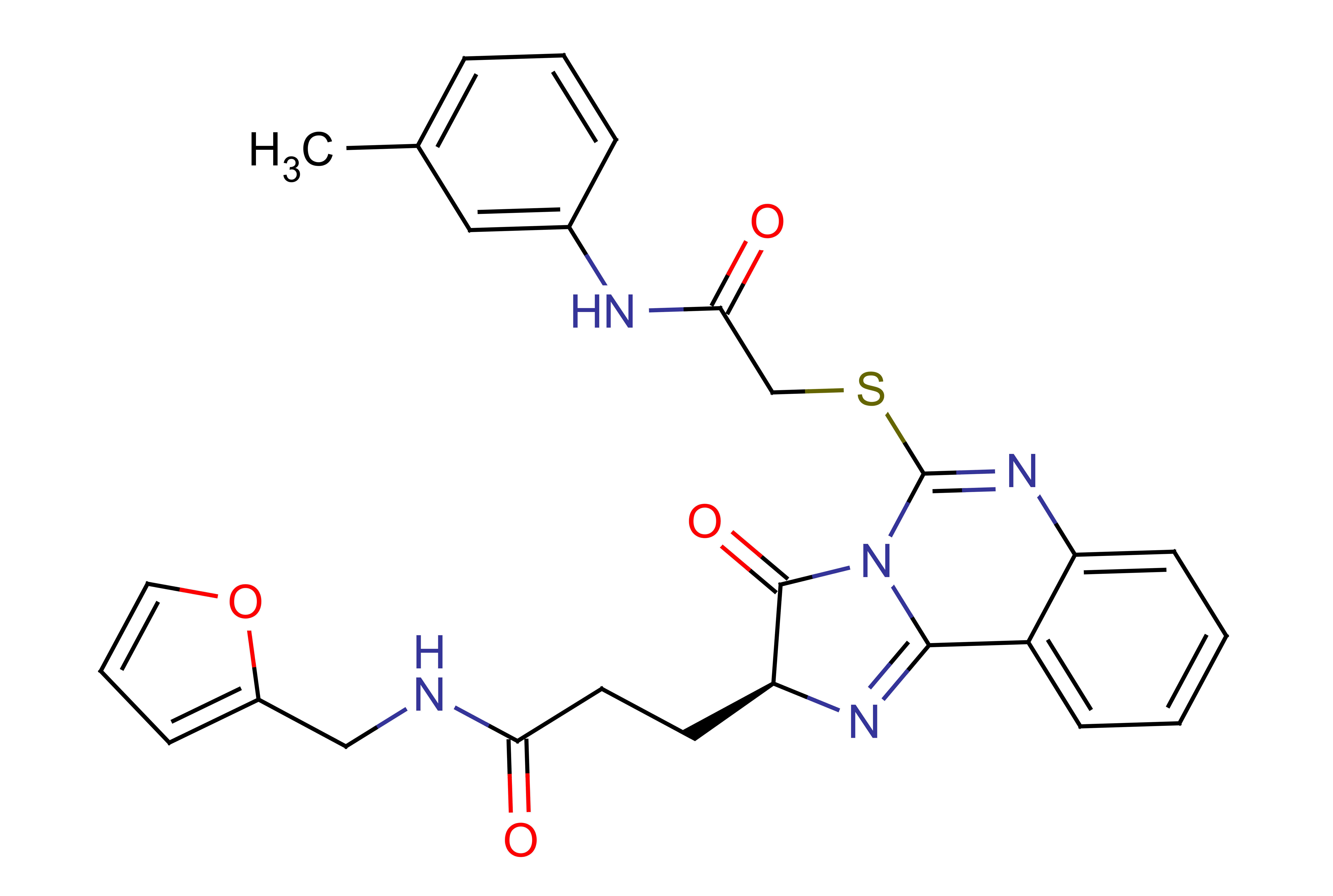 | 6.9 ± 1.8 | 52.8 ± 14 | 40.5 ± 1.3 | 51.0 ± 3.8 |
| Neq24 | 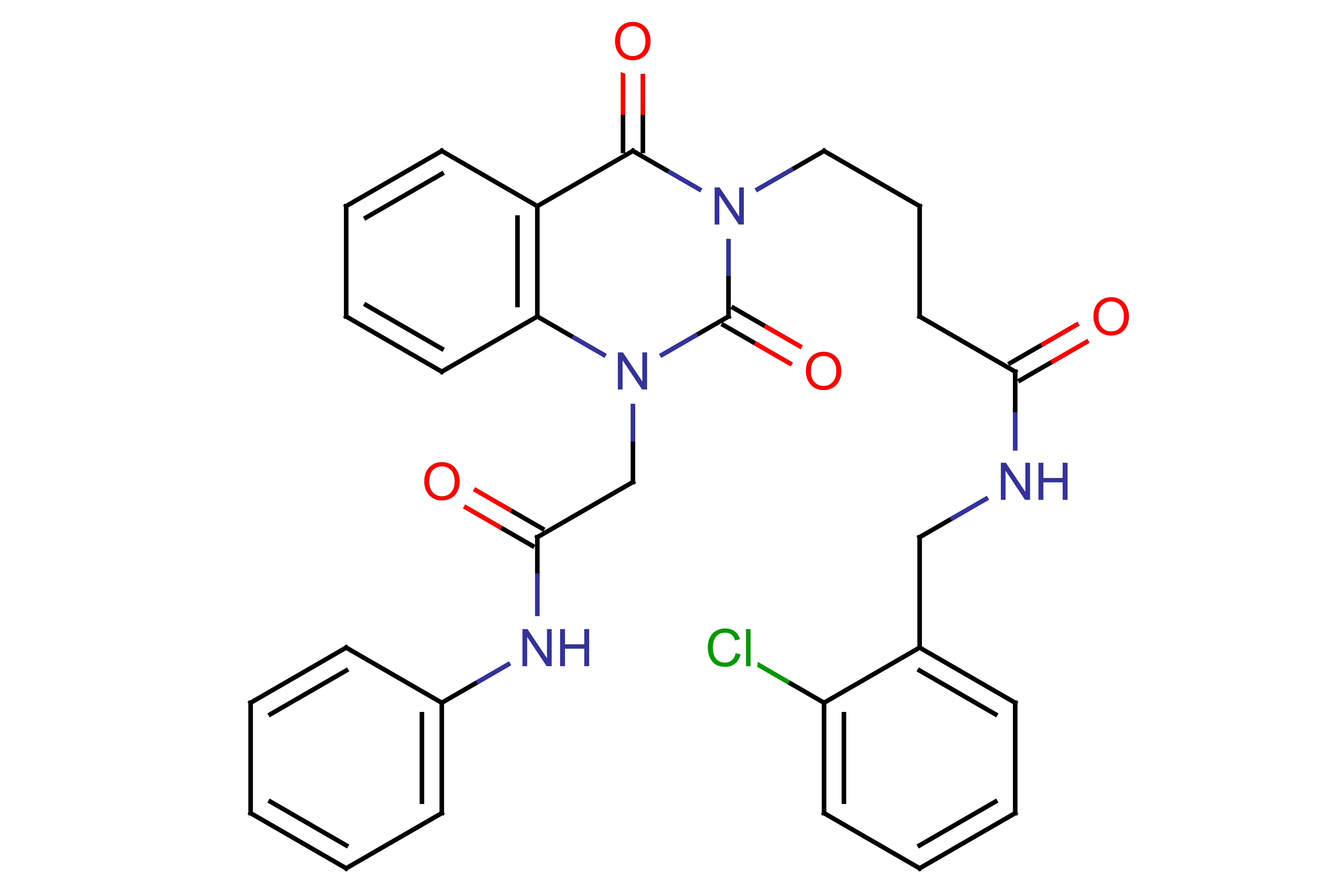 | 16.5 ± 1.7 | 3.80 ± 0.7 | 9.4 ± 1.2 | 8.6 ± 1.1 |
| Neq42 | 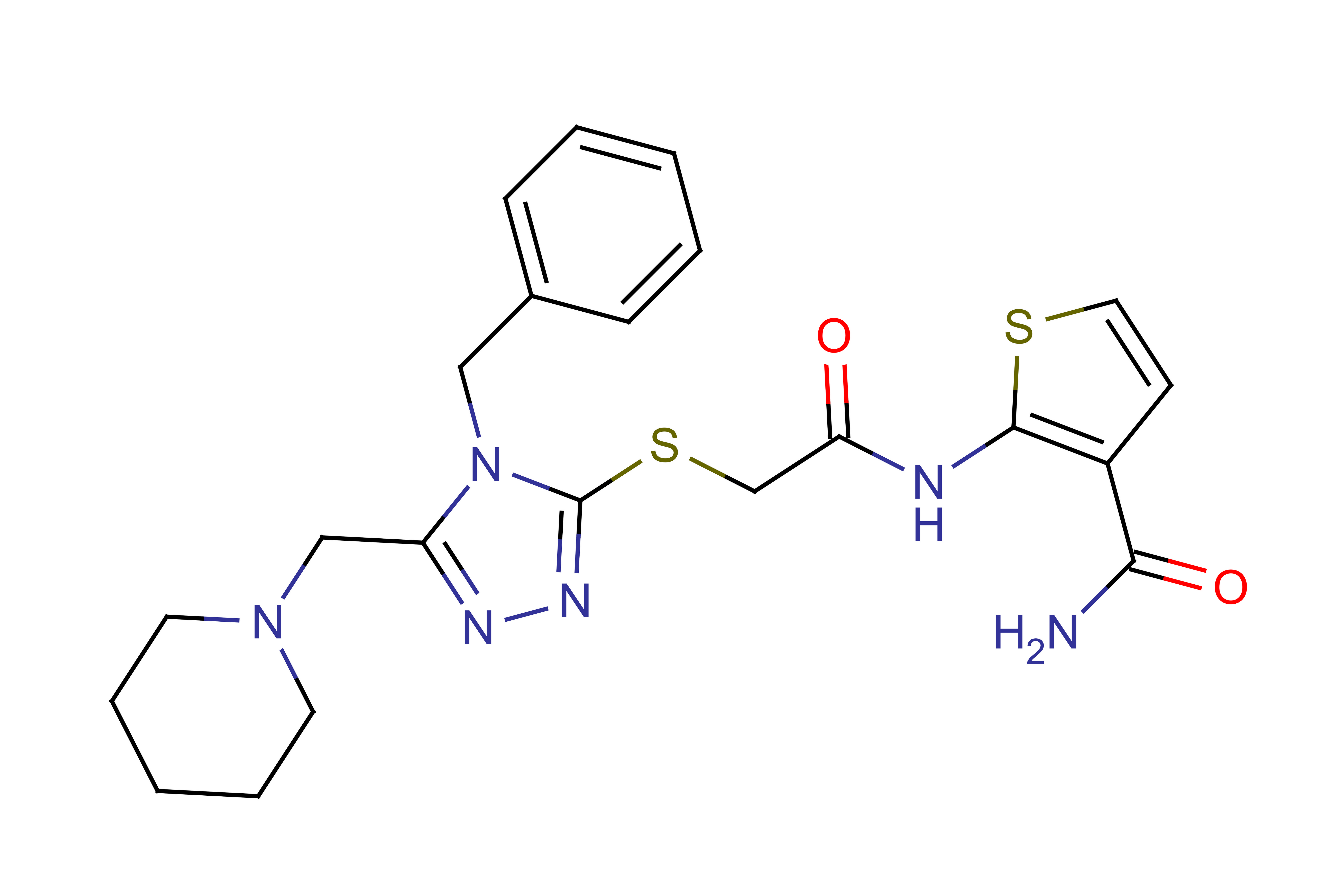 | 18.5 ± 5.5 | 35.6 ± 8.2 | 21.6 ± 5.4 | 35.1 ± 11 |
| Neq37 | 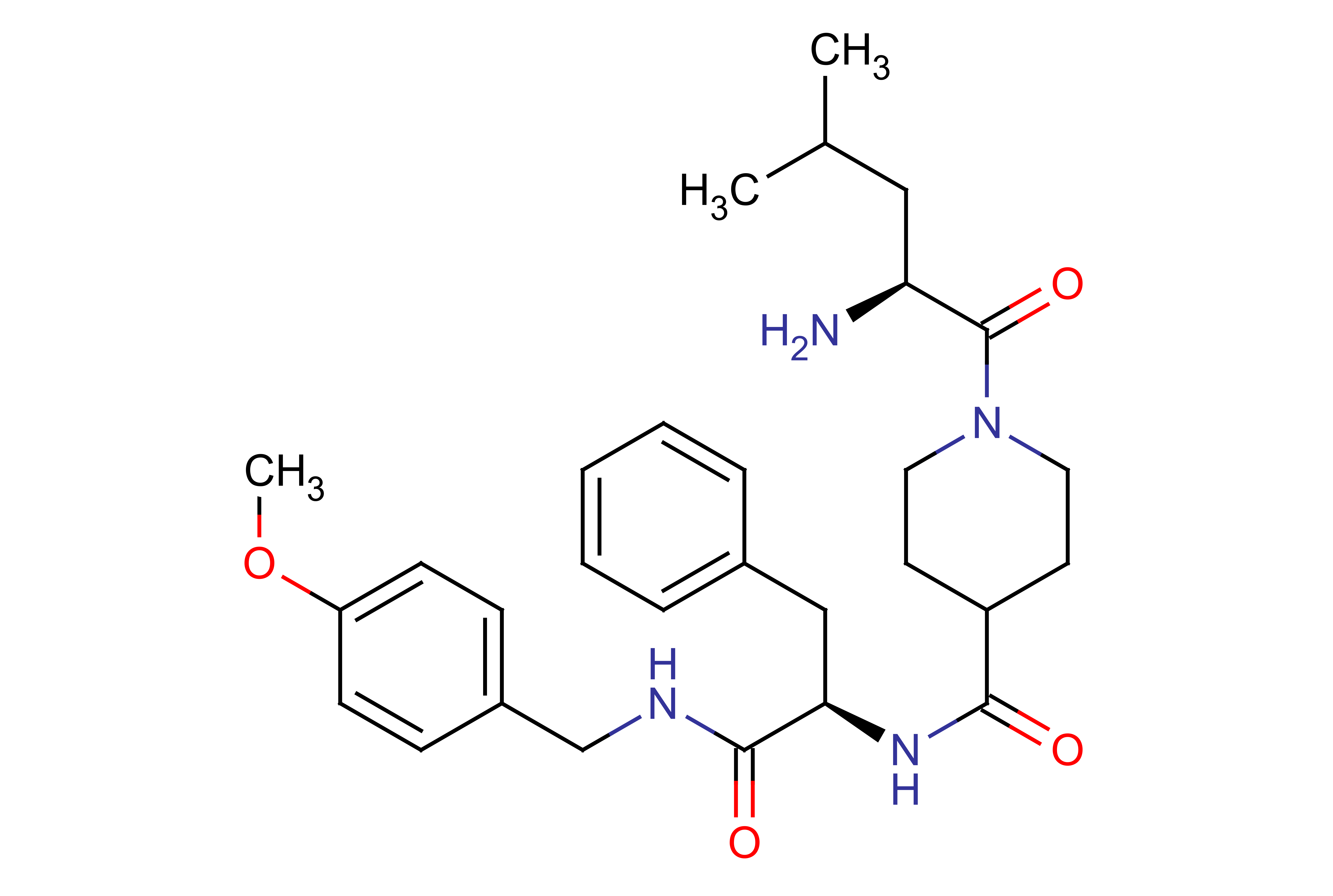 | 26.3 ± 3.1 | 41.1 ± 14 | 73.9 ± 3.2 | 81.5 ± 9.3 |
| Neq36 | 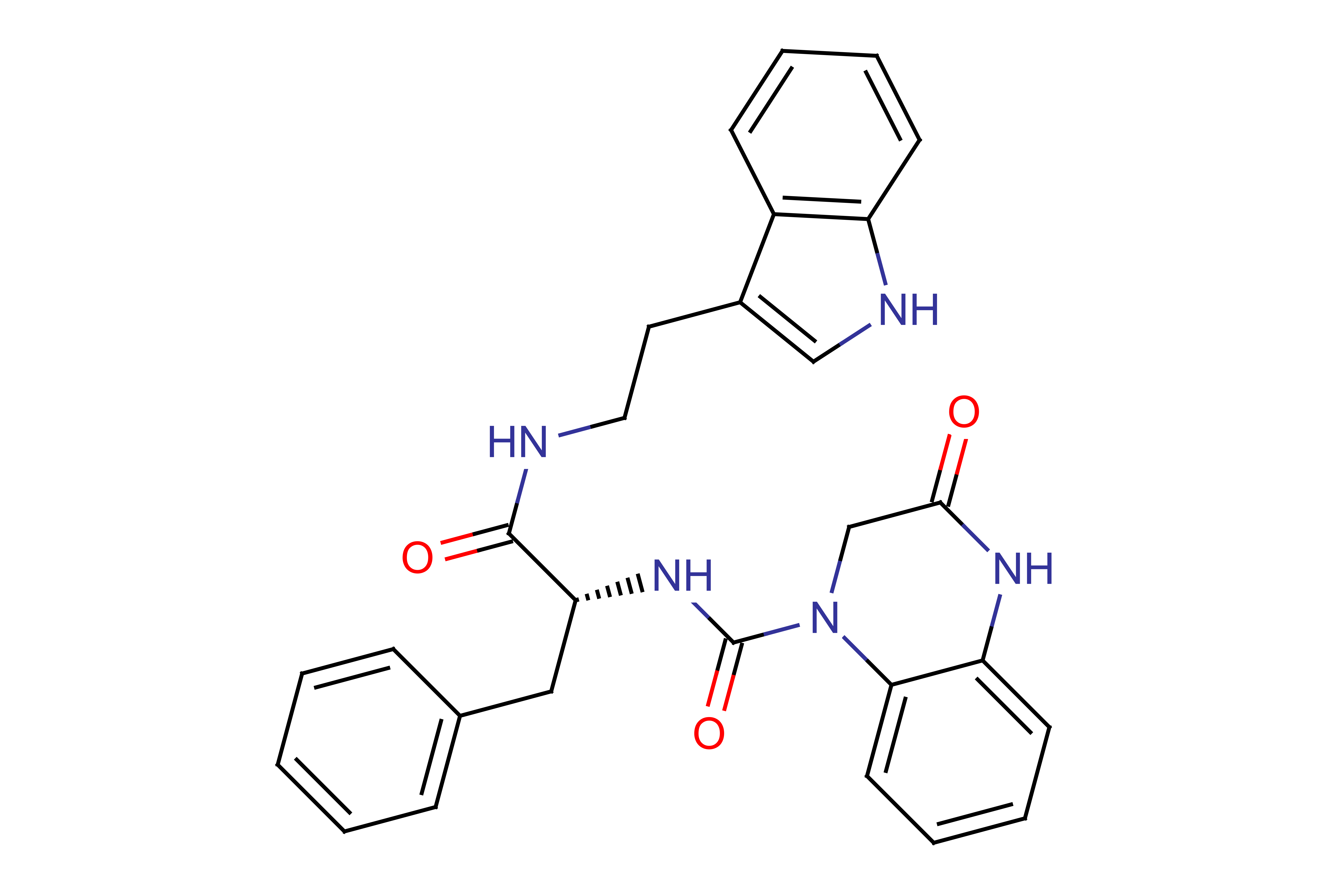 | 30.2 ± 3.7 | 19.1 ± 1.1 | 48.3 ± 9.2 | 20.4 ± 4.9 |
| Neq29 | 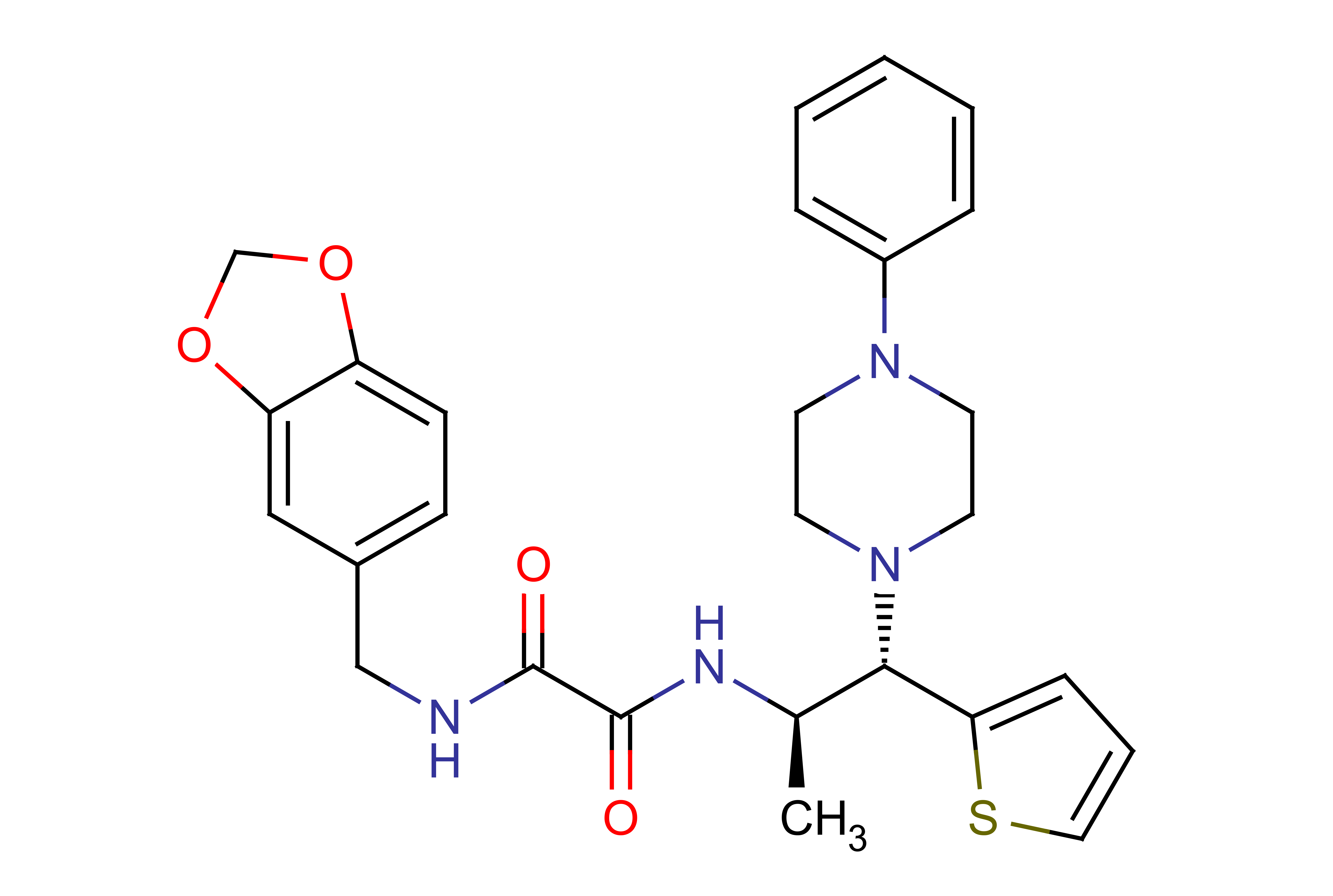 | 34.3 ±3.4 | 15.1 ± 4.3 | 17.4 ± 6.6 | 6.6 ± 1.1 |
| Neq41 | 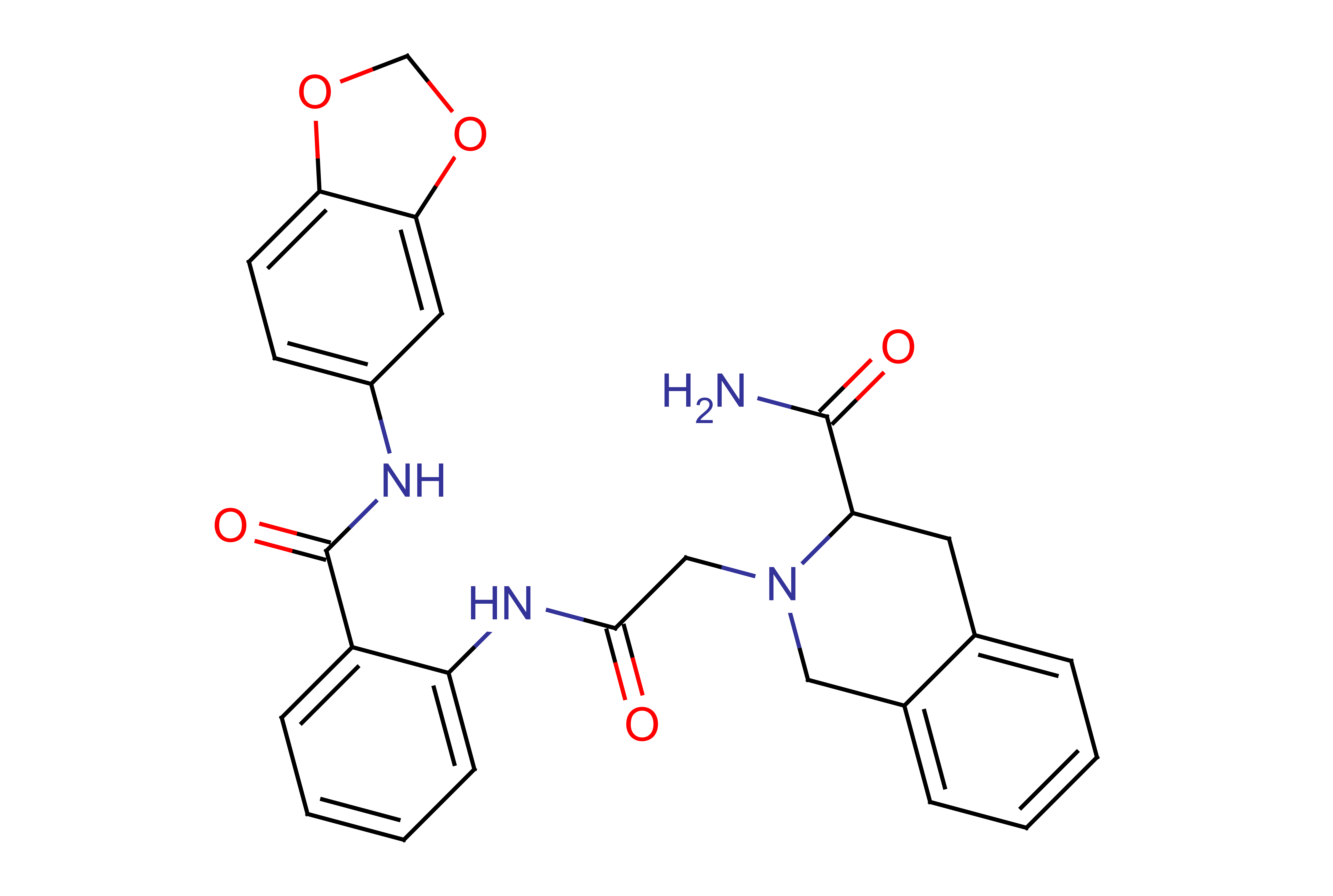 | 38.8 ± 4.1 | 89.1 ± 15 | 63.1 ± 14 | 92.1 ± 16 |
| Neq33 | 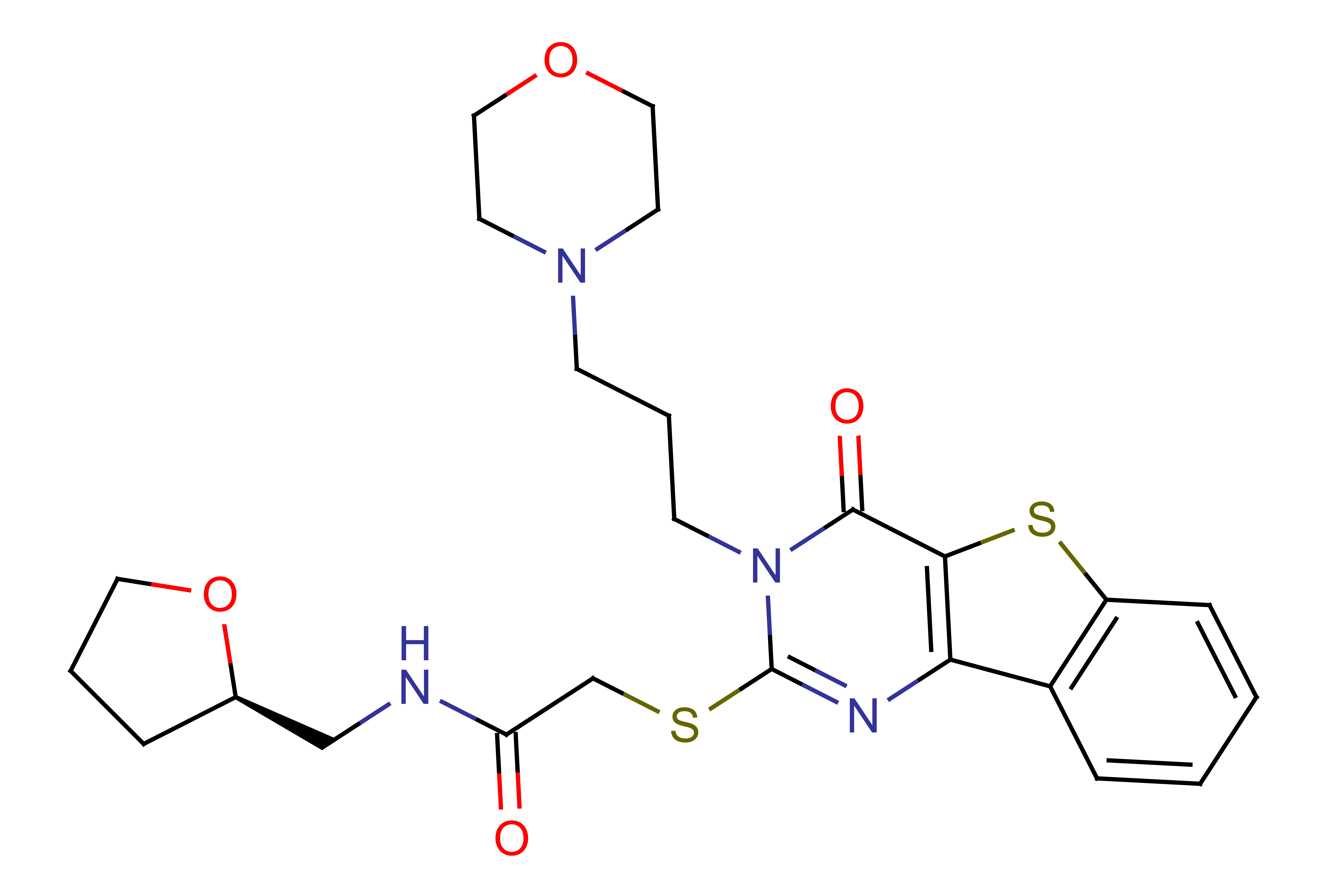 | 40.1 ± 1.2 | 67.6 ± 6.6 | 73.9 ± 6.3 | 81.3 ± 8.8 |
| Neq32 | 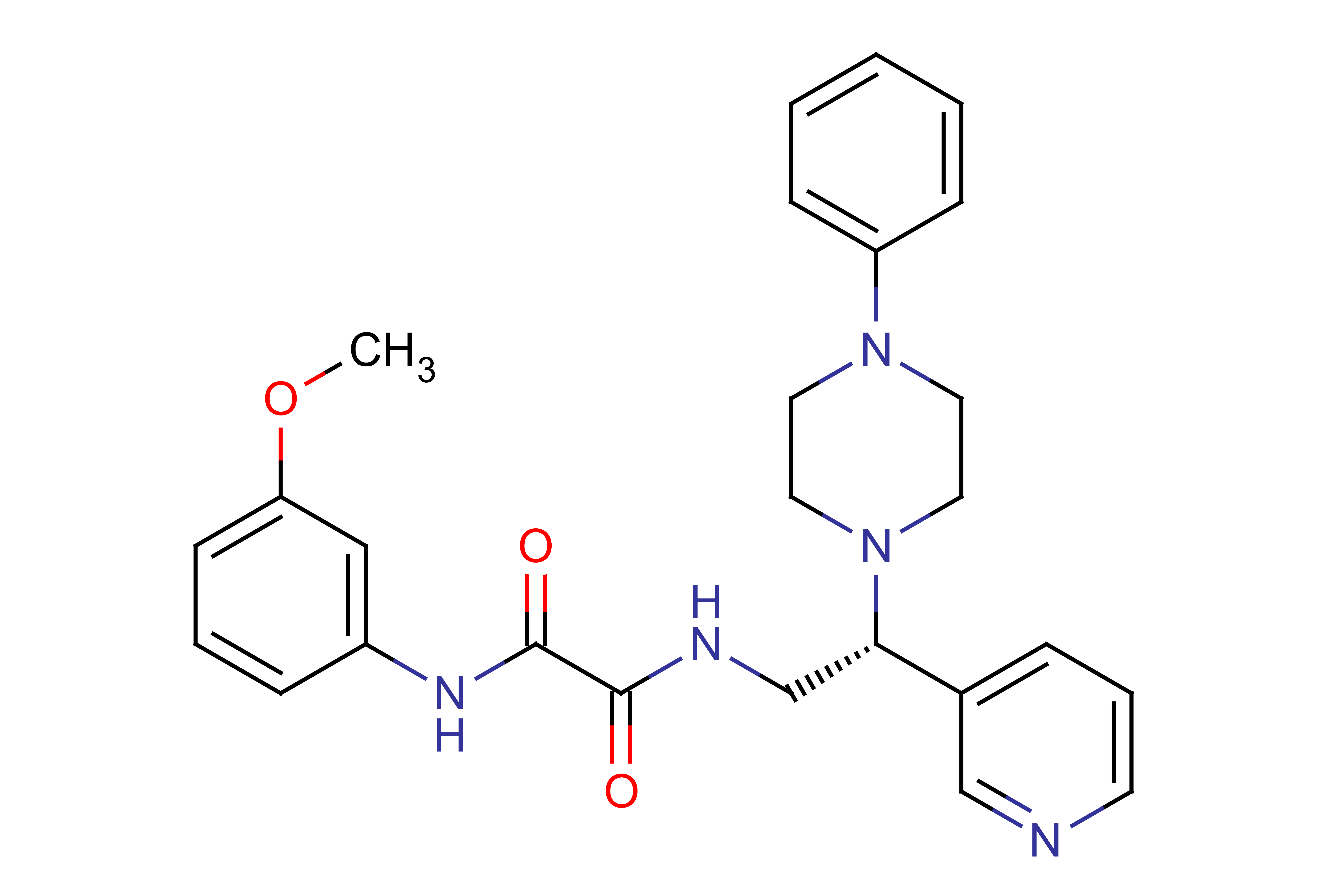 | 50.6 ± 8.3 | 44.4 ± 1.8 | 58.7 ± 8.3 | 51.8 ± 9.8 |
| Neq26 | 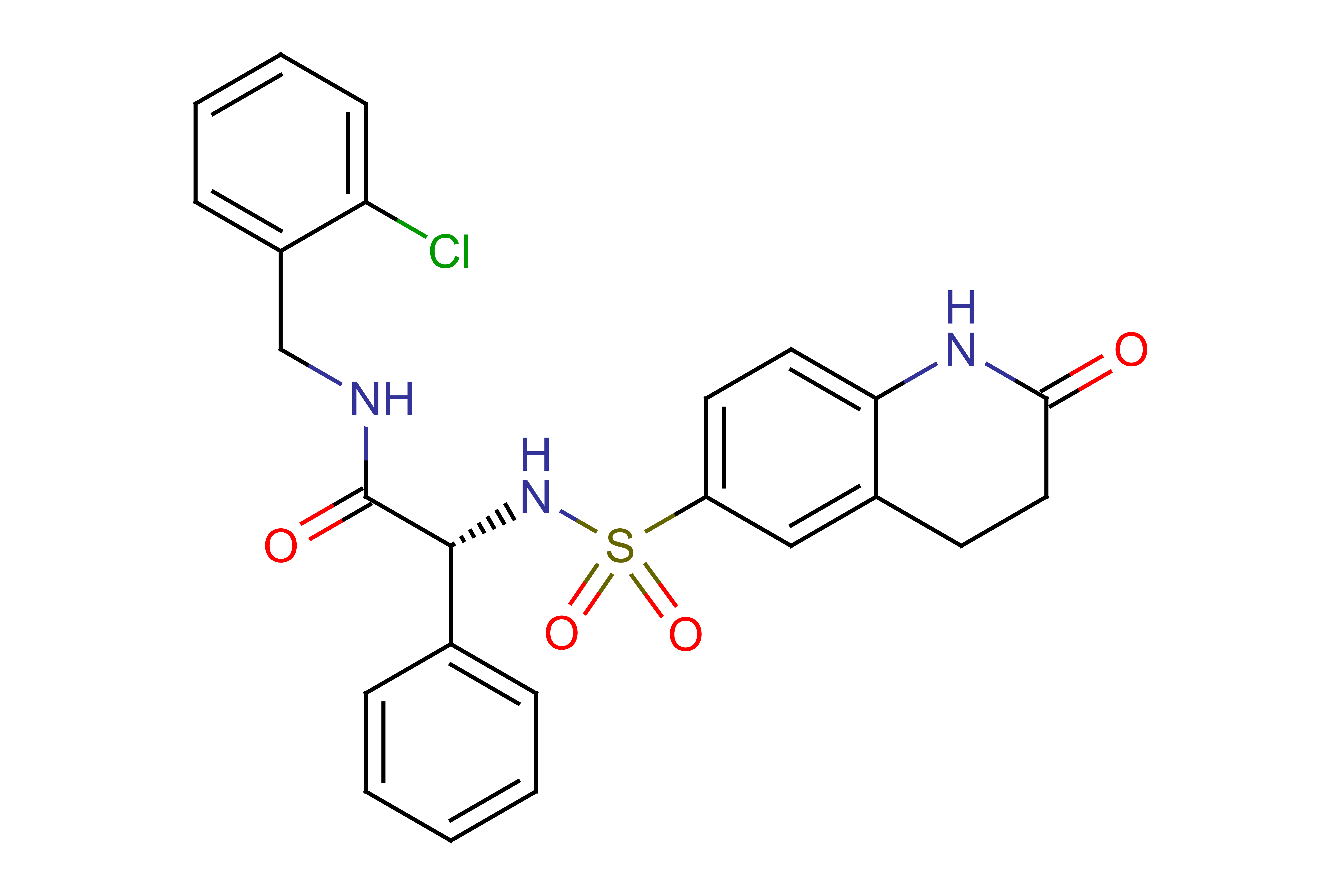 | 64.5 ± 9.0 | 63.4 ± 14 | 63.2 ± 1.1 | 50. 9 ± 2.2 |
| Neq21 | 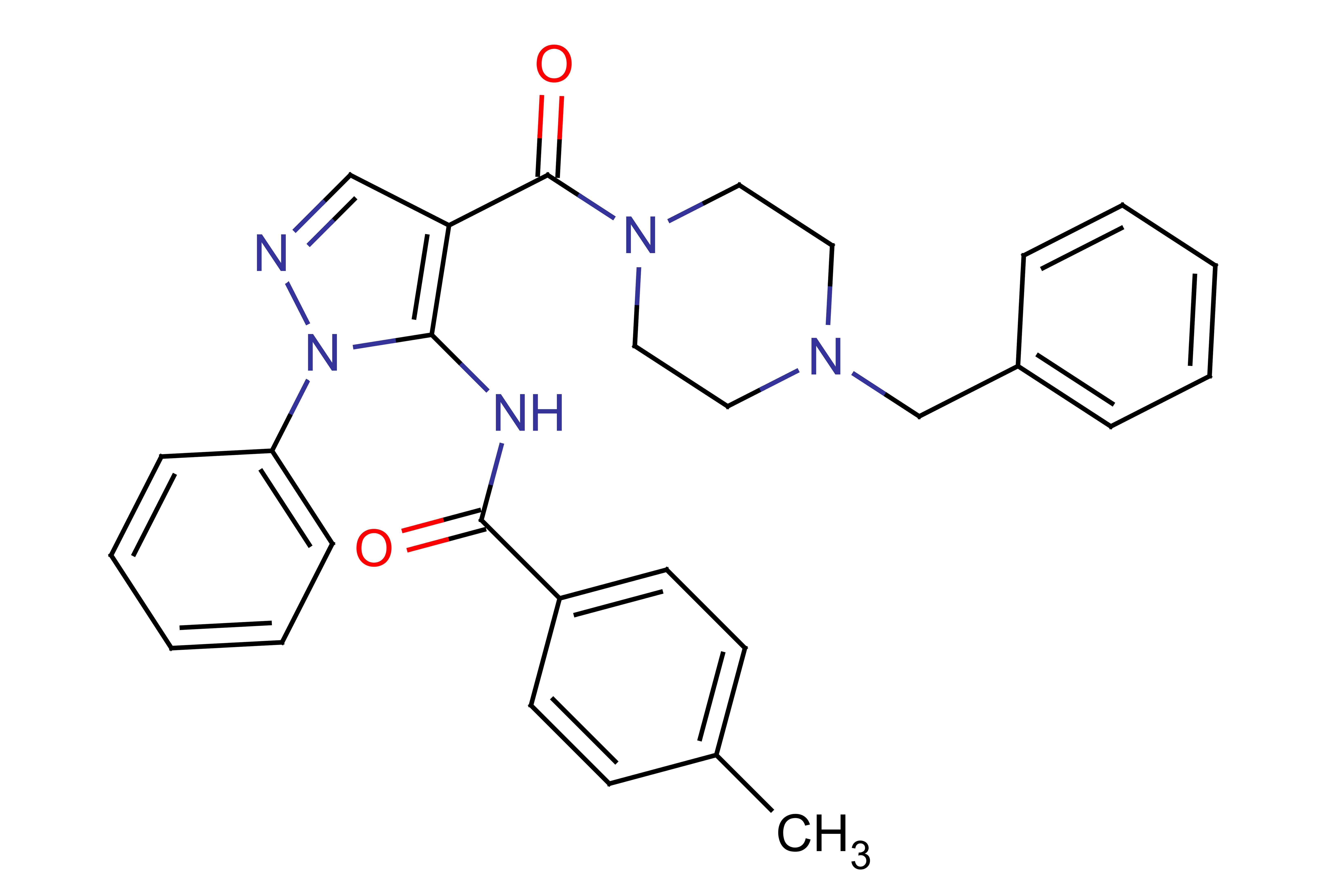 | inactive |  |  |  |
| Neq22 | 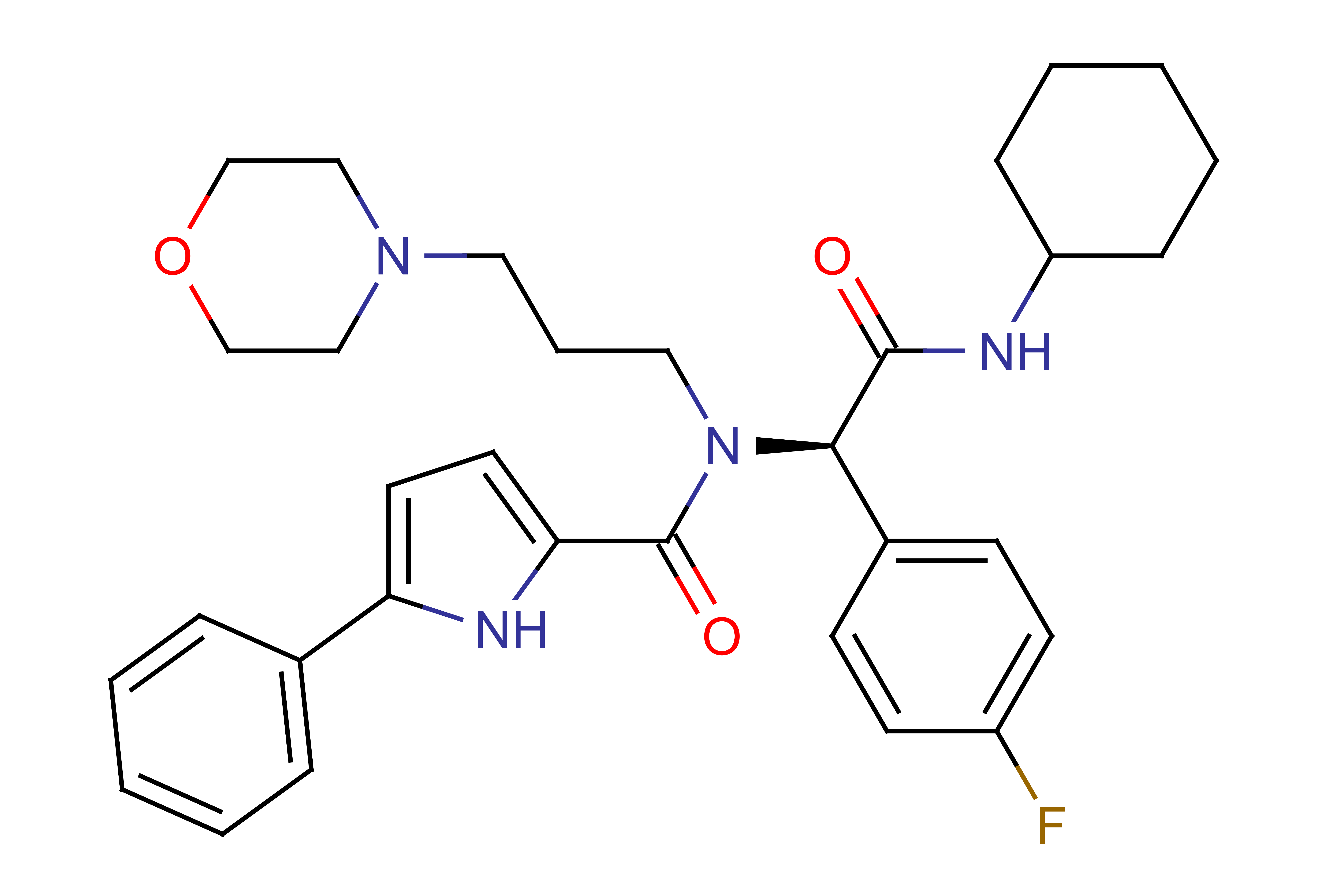 | inactive |  |  |  |
| Neq23 | 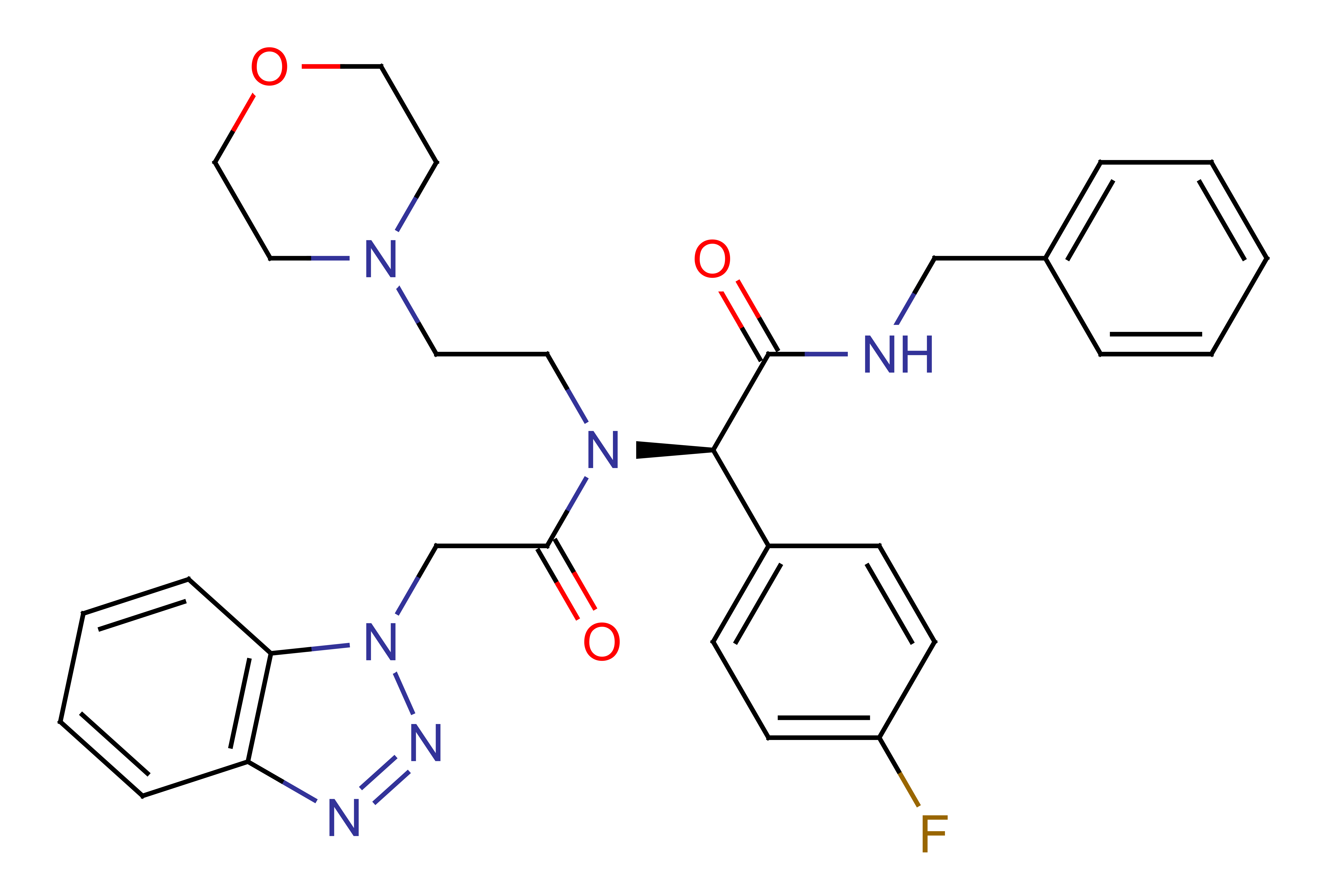 | inactive |  |  |  |
| Neq27 | 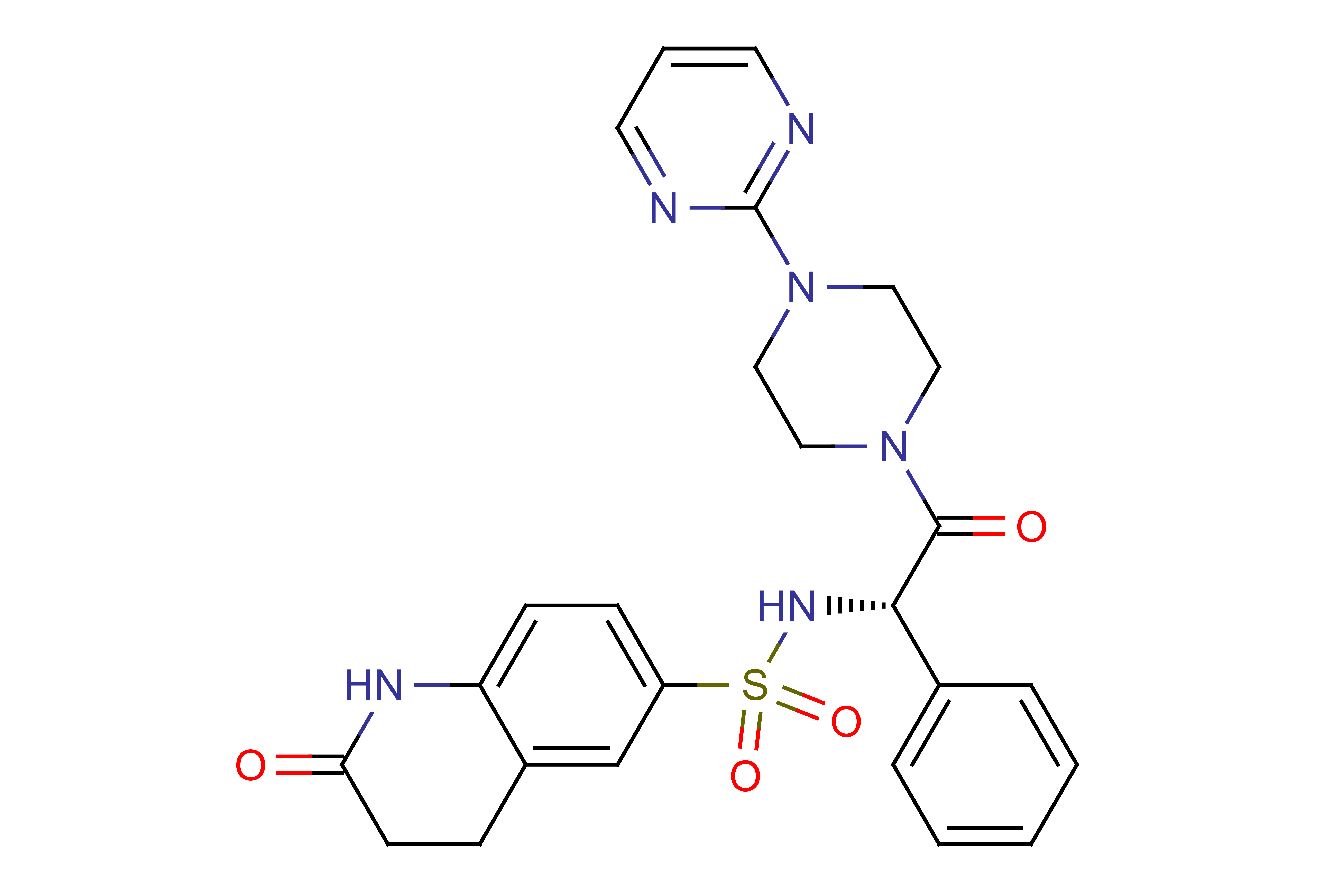 | inactive |  |  |  |
| Neq28 | 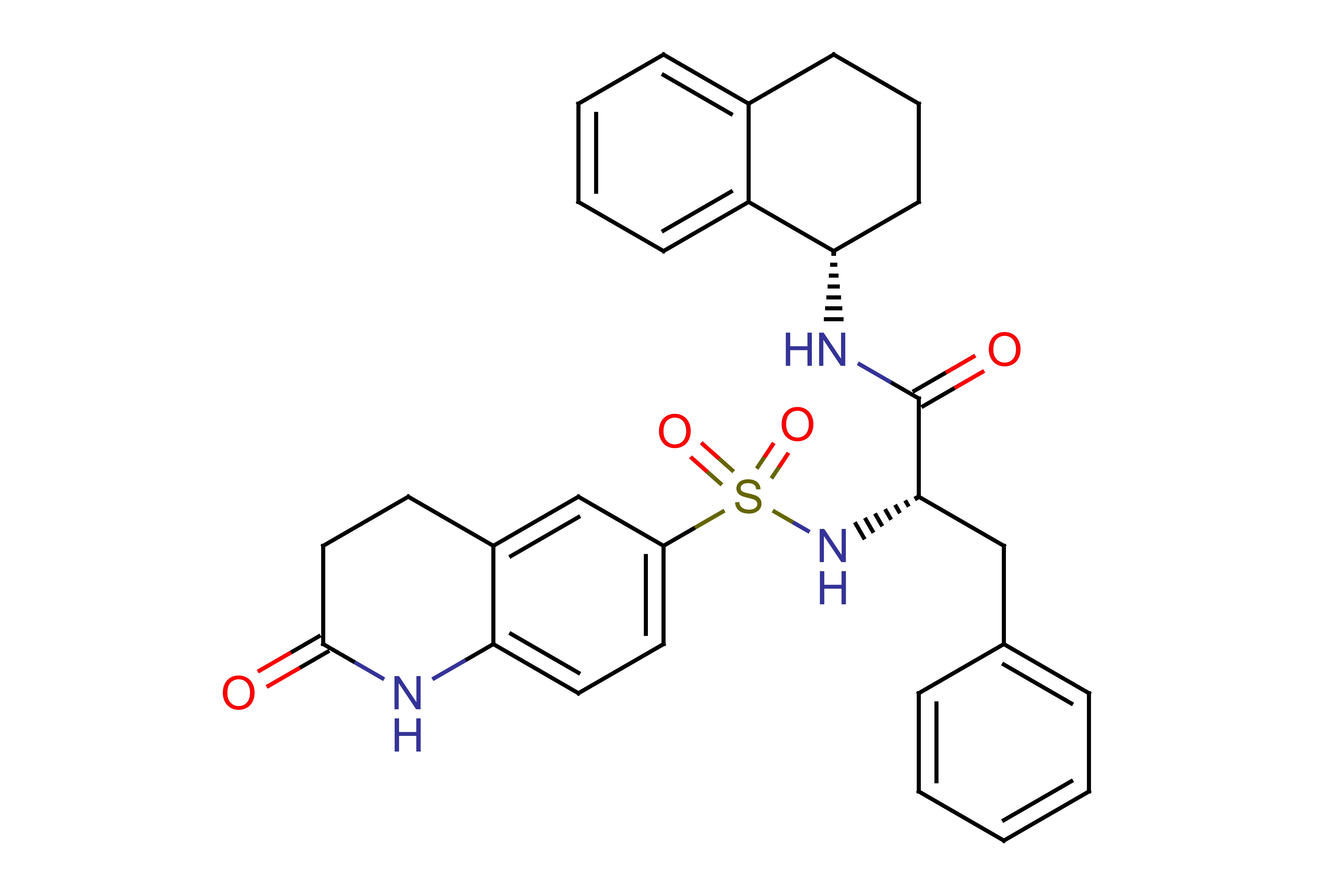 | inactive |  |  |  |
| Neq34 | 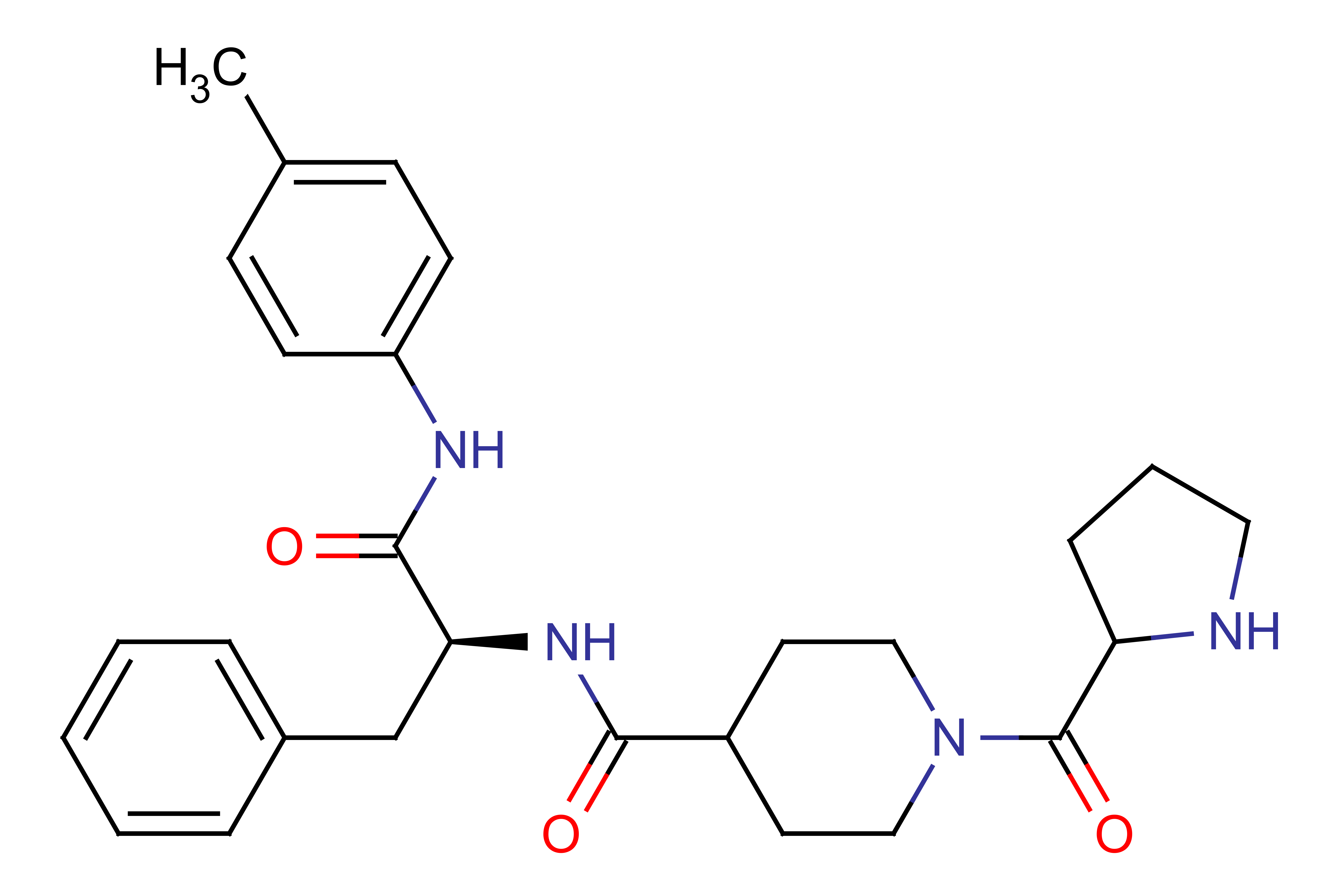 | inactive |  |  |  |
| Neq35 | 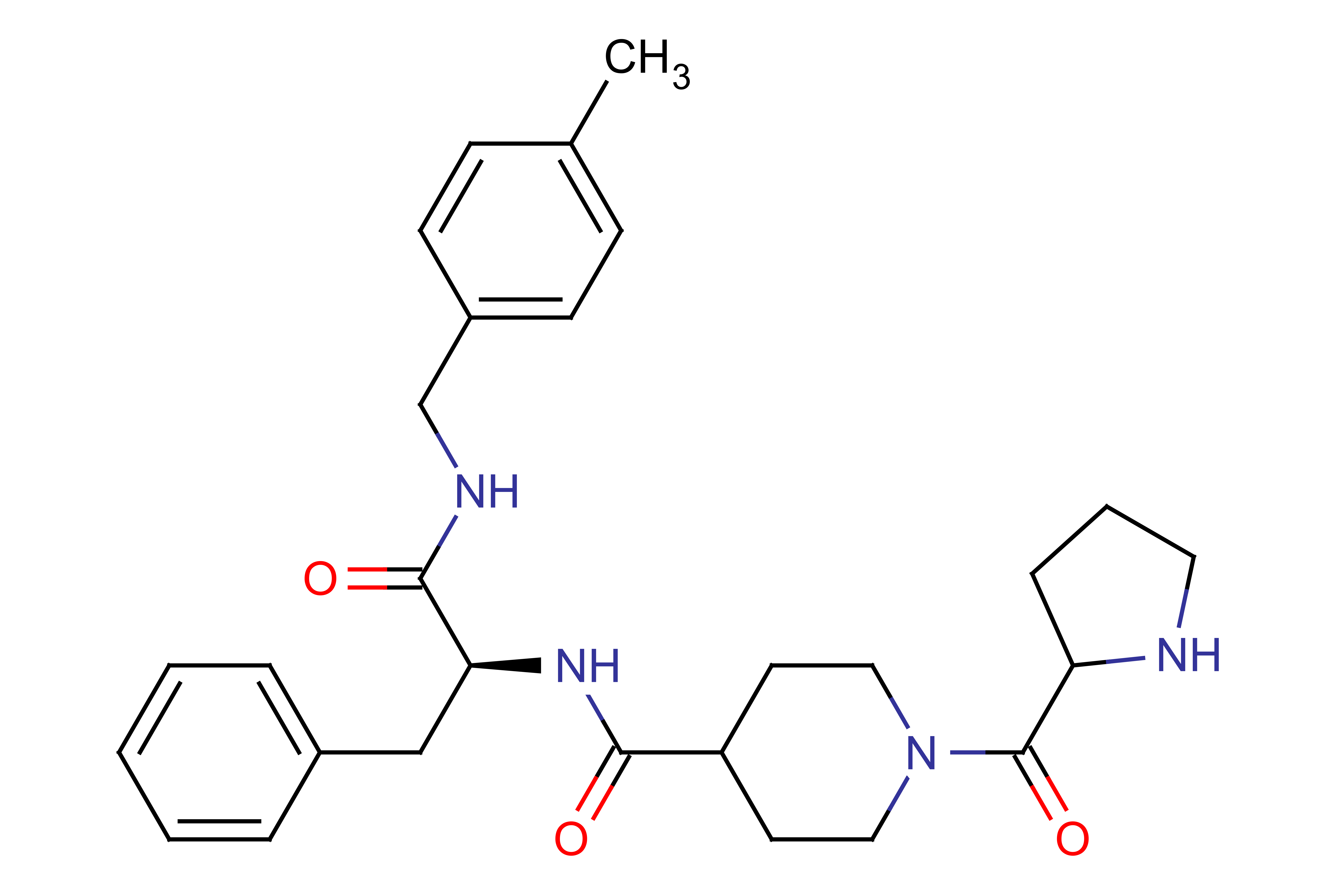 | inactive |  |  |  |
| Neq39 | 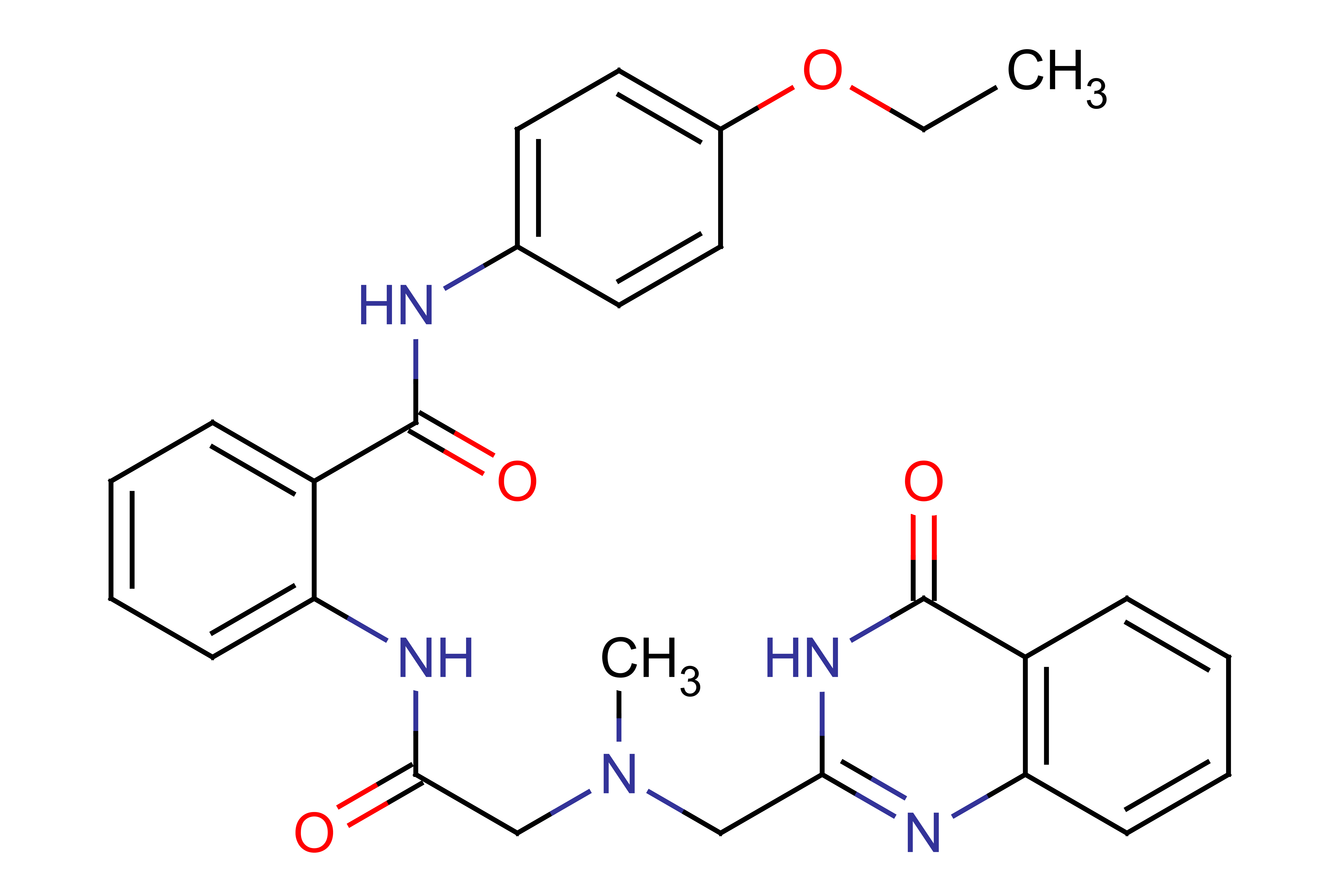 | inactive |  |  |  |
| Neq40 | 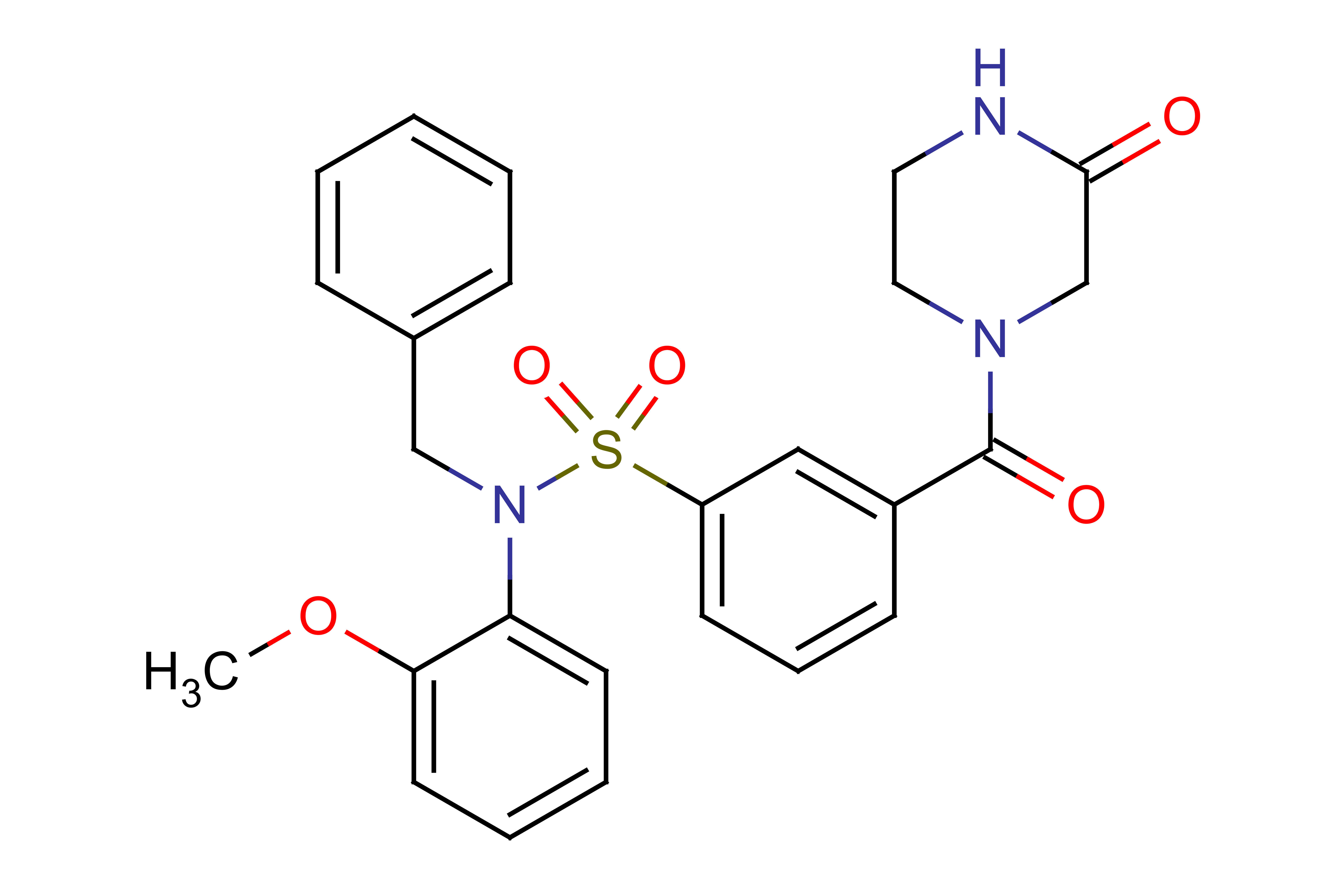 | inactive |  |  |  |
| Neq31 | 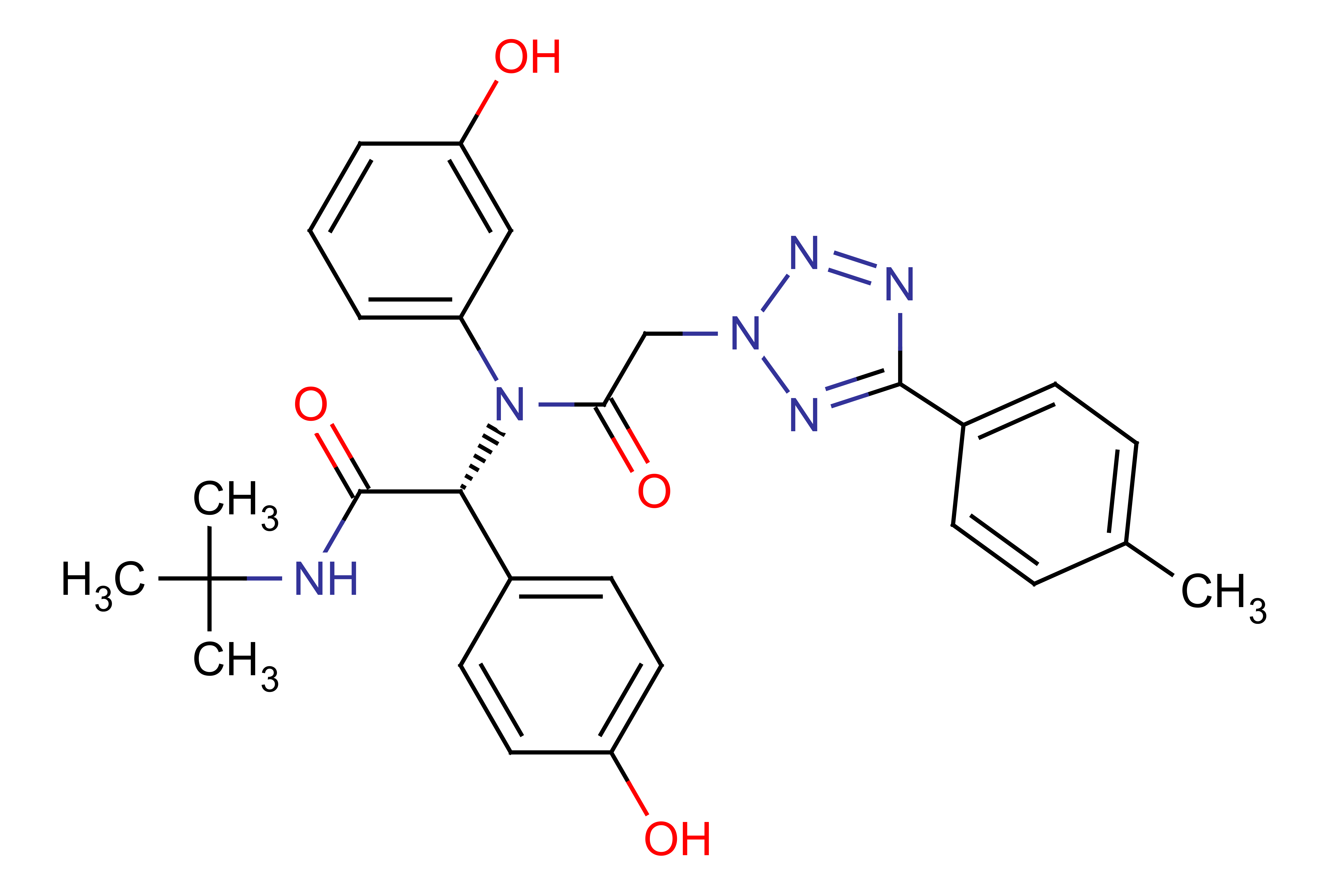 | inactive |  |  |  |
| Neq43 | 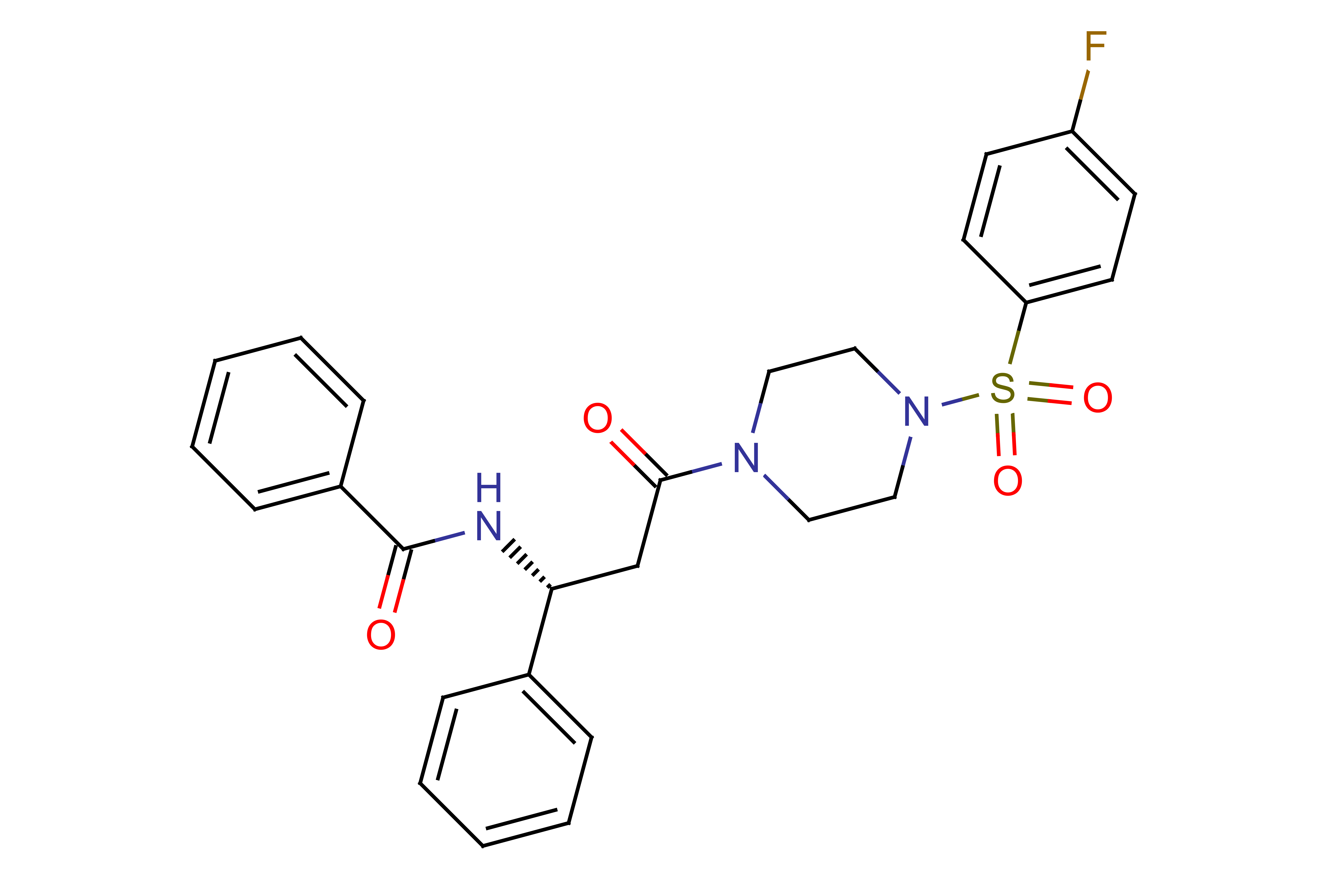 | inactive |  |  |  |
